# Bacterial tRNA 2′-O-methylation is dynamically regulated under stress conditions and modulates innate immune response

**DOI:** 10.1093/nar/gkaa1123

**Published:** 2020-12-04

**Authors:** Adeline Galvanin, Lea-Marie Vogt, Antonia Grober, Isabel Freund, Lilia Ayadi, Valerie Bourguignon-Igel, Larissa Bessler, Dominik Jacob, Tatjana Eigenbrod, Virginie Marchand, Alexander Dalpke, Mark Helm, Yuri Motorin

**Affiliations:** Université de Lorraine, CNRS, IMoPA (UMR7365), F54000 Nancy, France; Institute of Pharmaceutical and Biomedical Science, Johannes Gutenberg-University Mainz, 55128 Mainz, Germany; Institute of Medical Microbiology and Hygiene, Technische Universität Dresden, 01307 Dresden, Germany; Department of Infectious Diseases, Medical Microbiology and Hygiene, Ruprecht-Karls University Heidelberg, 69117 Heidelberg, Germany; Université de Lorraine, CNRS, IMoPA (UMR7365), F54000 Nancy, France; Université de Lorraine, CNRS, INSERM, IBSLor (UMS2008/US40), Epitranscriptomics and RNA Sequencing Core Facility, F54000 Nancy, France; Université de Lorraine, CNRS, IMoPA (UMR7365), F54000 Nancy, France; Université de Lorraine, CNRS, INSERM, IBSLor (UMS2008/US40), Epitranscriptomics and RNA Sequencing Core Facility, F54000 Nancy, France; Institute of Pharmaceutical and Biomedical Science, Johannes Gutenberg-University Mainz, 55128 Mainz, Germany; Institute of Pharmaceutical and Biomedical Science, Johannes Gutenberg-University Mainz, 55128 Mainz, Germany; Department of Infectious Diseases, Medical Microbiology and Hygiene, Ruprecht-Karls University Heidelberg, 69117 Heidelberg, Germany; Université de Lorraine, CNRS, INSERM, IBSLor (UMS2008/US40), Epitranscriptomics and RNA Sequencing Core Facility, F54000 Nancy, France; Institute of Medical Microbiology and Hygiene, Technische Universität Dresden, 01307 Dresden, Germany; Institute of Pharmaceutical and Biomedical Science, Johannes Gutenberg-University Mainz, 55128 Mainz, Germany; Université de Lorraine, CNRS, IMoPA (UMR7365), F54000 Nancy, France; Université de Lorraine, CNRS, INSERM, IBSLor (UMS2008/US40), Epitranscriptomics and RNA Sequencing Core Facility, F54000 Nancy, France

## Abstract

RNA modifications are a well-recognized way of gene expression regulation at the post-transcriptional level. Despite the importance of this level of regulation, current knowledge on modulation of tRNA modification status in response to stress conditions is far from being complete. While it is widely accepted that tRNA modifications are rather dynamic, such variations are mostly assessed in terms of total tRNA, with only a few instances where changes could be traced to single isoacceptor species. Using *Escherichia coli* as a model system, we explored stress-induced modulation of 2′-O-methylations in tRNAs by RiboMethSeq. This analysis and orthogonal analytical measurements by LC-MS show substantial, but not uniform, increase of the Gm18 level in selected tRNAs under mild bacteriostatic antibiotic stress, while other Nm modifications remain relatively constant. The absence of Gm18 modification in tRNAs leads to moderate alterations in *E. coli* mRNA transcriptome, but does not affect polysomal association of mRNAs. Interestingly, the subset of motility/chemiotaxis genes is significantly overexpressed in ΔTrmH mutant, this corroborates with increased swarming motility of the mutant strain. The stress-induced increase of tRNA Gm18 level, in turn, reduced immunostimulation properties of bacterial tRNAs, which is concordant with the previous observation that Gm18 is a suppressor of Toll-like receptor 7 (TLR7)-mediated interferon release. This documents an effect of stress induced modulation of tRNA modification that acts outside protein translation.

## INTRODUCTION

After transcription, all cellular RNAs enter into complex post-transcriptional maturation processes, including a multitude of enzymatic modifications altering the chemical properties of parental nucleotides. Over 170 different chemical modifications have been described so far (see http://modomics.genesilico.pl/ (1)), with the most common RNA modifications being pseudouridylation (U-to-Ψ conversion) and various types of base and ribose methylations. Among all RNA types, transfer RNAs (tRNAs) show the highest density of modifications which have multiple cellular functions. In addition to purely structural roles and general protection of RNA molecules from degradation (by alkaline hydrolysis and/or endonucleolytic enzymes (reviewed in (2–6)), tRNA modifications increase ribosome binding affinity, reduce reading errors and modulate frameshifting, thus affecting mRNA translation rate and fidelity (5,7–10).

Recently, the dynamic behavior of eukaryotic tRNA modifications has been linked to regulation of cell growth or cellular stress responses (11–13). The majority of such studies has been conducted in lower eukaryotes, where it was demonstrated that tRNA modification profile changes upon elevated growth temperatures or under growth arrest conditions (14–17). By quantification of tRNA modifications by LC-MS/MS it was demonstrated that levels of Cm, m^5^C and m^2^_2_G modifications in *Saccharomyces cerevisiae* tRNAs increase after exposure to oxidative stress induced by hydrogen peroxide (H_2_O_2_), but not in response to hypochlorite (NaOCl). Moreover, levels of 23 out of the 25 analyzed *S. cerevisiae* tRNA modifications change in response to toxic substances, such as NaAsO_2_ (sodium arsenite, toxicity) or methyl methanesulfonate (CH_3_-SO_3_-CH_3_, DNA damage) (18). These observations clearly demonstrate the importance of selected tRNA modifications in adaptive response to toxic substances. In addition, the wobble-base tRNA modifications (pos 34) modulate regulation of a codon-biased translation during cellular stress response (19–21). Overall, the emerging concept is that dynamically regulated tRNA modifications allow regulation of gene expression at the translational level, by differential usage of redundant mRNA codons, to provide better adaptation to stress conditions by the expression of crucial stress-related proteins (22–25).

In bacteria and archaebacteria (26), correlations were reported between tRNA modification profiles on one hand and growth temperature and oxidative stress on the other hand (27–32). However, only few studies link cellular adaptation response to stress conditions to dynamic changes of specific tRNAs and provide a molecular basis for the impact of modification levels on gene expression. For instance, MiaB-mediated ms^2^i^6^A37 modification in *Escherichia coli* tRNA plays a role in stress response by modulating the steady-state level of the stress-induced transcription factor RpoS (33). Moreover, a study on *Mycobacterium bovis* demonstrated that early hypoxia induces wobble modification cmo^5^U in tRNA^Thr^ (cmo^5^UGU), thus leading to codon-biased translation (34).

RNA 2′-O-methylation is the most common modification occurring at the ribose moiety. In tRNAs, synthesis of such residues is typically mediated by position-specific, stand-alone, proteinaceous tRNA methyltransferases (MTases) that catalyze the transfer of a CH_3_-group (Me-group) from a methyl donor, generally S-adenosyl-L-methionine (SAM or AdoMet) to the tRNA (35). Only three positions are known to be 2′-O-methylated in *E. coli* tRNAs. The major site of tRNA 2′-O-methylation is located in the D-loop at position 18, where the highly conserved G residue is frequently converted to a 2′-O-methylated guanine (Gm) by TrmH (SpoU)-like enzymes. This modification is quite common and is present in 12 out of 35 *E. coli* tRNAs. Two other Nm residues are located in the anticodon loop at positions 32 and 34 (wobble position), where Cm and Um/cmnm5Um residues are present in 8 selected tRNAs. TrmJ and TrmL enzymes are responsible for 2′-O-methylation of Cm/Um32 and Cm/cmnm5Um34, respectively (reviewed in (36)).

tRNA 2′-O-methylation in *E. coli* (Gm18) is also known to be an effective structural anti-determinant for recognition by the mammalian innate immune system via interaction with Toll-like receptors (TLR7) (37–41). Innate immune system receptors recognize microbial-associated molecular patterns (MAMPs) and activate immune cells. In the TLR receptor family, TLR7 and TLR8 are activated by single stranded RNA of bacterial origin, which typically contains significantly fewer modifications than mammalian rRNA and tRNA. In humans, TLR7 is preferentially expressed in plasmacytoid dendritic (pDC) and B cells and specifically induces the production of type I interferon such as IFN-α, whereas TLR8 is found in myeloid dendritic cells and regulatory T cells and its activation leads to the production of other inflammatory cytokines (for reviews (42,43)). Next to spatial restrictions and sequence positions in nucleic acids, ribose-methylated guanosine in tRNA (Gm18) was among the first RNA modifications to be identified as crucial in the distinction between ‘self’ and ‘non-self’ RNA molecules by the innate immune system (44). *In vitro* studies showed that incorporation of a 2′-O-methylation in synthetic RNA transcripts, siRNA (45) or 18S rRNA prevents TLR activation (40,46). Given that mammalian tRNA and rRNA, which together amount to over 90% of cellular RNA mass, contain high amounts of ribose-methylated nucleosides, the current hypothesis views ribose methylation, in particular Gm, as a positive identifier of ‘self’ i.e. human/mammalian RNA. In line with this, it was demonstrated that native bacterial *E. coli* tRNA^Tyr^, which is one of Gm18 containing tRNA, actually antagonizes TLR7-mediated signaling and the presence of this modification actually suppresses bacterial tRNA recognition (37–38,41), defining a potential principle of immune escape.

In this study we focused on stress-induced modulation of tRNA 2′-O-methylations in a model bacteria *E. coli*. Stress conditions were, in part, mimicking the host environment during bacterial invasion: mild antibiotic stress and limited nutrient supply (starvation). We demonstrate that these stimuli indeed modulate global profile of *E. coli* tRNA 2′-O-methylation thus pointing out their potential role in the escape from the host innate immune system. Reliable measurements of 2′-O-methylation levels were performed by a combination of two orthogonal techniques: high-throughput sequencing-based RiboMethSeq (47–49) and LC-MS/MS analyses (50–52). Data obtained by both techniques demonstrate an excellent correlation for technical and biological replicates. Under stress conditions (mainly starvation and mild sub-lethal antibiotic stress) Gm18 methylation was observed in a majority of potentially modified tRNAs, while it is reduced under standard laboratory growth conditions. Deletion of TrmH, responsible for Gm18 modification in tRNAs, only moderately alters *E.coli* mRNA transcriptome, and does not affect polysomal association of mRNAs. However, mutant ΔTrmH strain shows significant overexpression of motility/chemiotaxis genes, and, consequently, increased swarming motility. Moreover, stress also modulates immunostimulatory properties of bacterial RNA, as assessed by PBMC immunostimulation assay. Altogether, our data demonstrate that starvation and antibiotic stress conditions lead to a selective increase of Gm18 methylation in tRNA and, in turn, to the inhibition of the host immune response.

## MATERIALS AND METHODS

### Bacterial strain and stress conditions used

The *E. coli* strain DH5α (lacZ Delta M15 Delta(lacZYA-argF) U169 recA1 endA1 hsdR17(rK-mK+) supE44 thi-1 gyrA96 relA1) was grown in lysogeny broth (LB) medium to mid-log phase (0.8–1.2 OD_600nm_) at 37°C, 190 rpm for the control condition (LB 37°C).

Eleven different stress conditions were applied: LB 20°C, LB 42°C, minimal medium M9 37°C, M9 20°C, LB 37°C Hypoxia, LB 20°C Hypoxia, starvation in polymerase chain reaction (PBS), streptomycin, spectinomycin, chloramphenicol and gentamycin in sub-lethal concentrations (details on bacterial cultures under stress conditions are given in Supplementary Data).

### tRNA extraction

Direct tRNA extraction from *E. coli* using TRIzol^™^ reagent was done essentially as described previously (53). RNA concentration was controlled by UV absorbance using NanoDrop 2000c (Thermo Fisher Scientific). The profile of isolated tRNA fractions was assessed by capillary electrophoresis using an RNA 6000 Pico chip on Bioanalyzer 2100 (Agilent Technologies).

### Isolation of individual tRNA species

The principle of individual tRNA isolation is outlined in the Supplementary Figure S1. Pure tRNA isoacceptors were isolated by affinity column chromatography on covalently immobilized fully complementary synthetic DNA oligonucleotide (54). Further details are given in Supplementary Table S1 and in the Supplementary Data.

### RiboMethSeq analysis of 2′-O-methylations in tRNAs

Analysis of *E. coli* tRNA 2′-O-methylation was performed according to tRNA-adapted version of general RiboMethSeq protocol as described previously (48,53). Further details are given in Supplementary Data.

### Analysis of modified nucleosides by LC-MS(MS)

Total tRNA or individual tRNA samples were digested and dephosphorylated into nucleosides, mixed with internal standard for quantification and analyzed with an Agilent 1260 Infinity system in combination with an Agilent 6460 triple quadrupole mass spectrometer with an electrospray ion source as described in Supplementary Table S2 (50,52,55).

### Polysome profiling

Analysis of polysome-associated mRNAs and tRNA was performed in triplicate, using WT and ΔTrmH isogenic *E. coli* strains from KEIO collection (56). Cells were grown to optical density OD_600nm_ of 0.6 in LB medium (supplemented by 25 μg/ml of kanamycin for ΔTrmH) at 37°C, collected by centrifugation and lysed by lysozyme and freeze-thaw cycles to obtain cellular extract. An aliquot was conserved to serve as a reference of total mRNA/tRNA expression (‘Lysate fraction’). Total extract was loaded onto sucrose gradient (5–40%) and polysomal fraction was collected after centrifugation at 35 000 rpm for 2.5 h at 4°C. RNA from polysomal fraction was extracted by TRI Reagent and used for further analysis (‘Polysome fraction’). Depletion of *E. coli* rRNAs and simultaneous fragmentation was done using QIAseq FastSelect–5S/16S/23S Kit (Cat No./ID: 335925). Resulting RNA fragments were 3′-dephosphorylated and 5′-phosphorylated and used for library preparation with NEBNext Small RNA kit (E7330). Sequencing was done on Illumina HiSeq1000 in SR50 mode. About 30–40 millions of raw reads were obtained for each sample. Analysis of rRNA mapped reads confirmed low residual level of rRNA fragments (∼5%). Raw reads were aligned to *E. coli* tRNA reference database followed by mapping to KEIO *E. coli* transcriptome. Counting was done independently for tRNAs and mRNA and normalized to library size. Differential expression analysis was done using DESeq2 R package (57).

### Immunostimulation assays

Human PBMCs were isolated from heparinized blood of healthy volunteers upon informed consent or from anonymized buffy coats from the local blood donation service. The study was approved by the local ethical committee (registration no. S-716/2017). After isolation by standard Ficoll-Hypaque density gradient centrifugation (Ficoll 1.078 g/ml), PBMCs were resuspended in RPMI 1640 supplemented with 2% heat-inactivated human serum and seeded in 96-well flat bottom plates at a cell number of 2 × 10^5^ cells/well. For the purification of plasmacytoid dendritic cells (pDCs) from isolated PBMCs, a negative selection program was performed using the autoMACS Pro Separator according to the manufacturer's protocol with the plasmacytoid dendritic cell isolation kit II (Miltenyi Biotec, Bergisch Gladbach, Germany). A purity of CD303/CD123 double positive cells of >90% was confirmed by FACS analysis. The pDCs were resuspended in RPMI 1640 supplemented with 5% heat inactivated human serum and seeded in 96-well flat bottom plates at a cell number of 5000 cells/well. For transfection experiments, RNAs were encapsulated with DOTAP at a ratio of 3 μl DOTAP per 1 μg of RNA in Opti-MEM reduced serum medium following the manufacturer's protocol. PBMCs or pDCs were then transfected with 1 μg/ml or 0.5 μg/ml RNA. After 20 h of incubation in a humidified 5% CO_2_ atmosphere at 37°C, cell-free supernatants were collected for cytokine quantification via ELISA (eBioscience, Frankfurt, Germany).

### Motility assay

The parental WT and the ΔTrmH *E. coli* strains (K-12 KEIO collection) were maintained on sheep blood agar plates at 4°C for a maximum of 14 days. The protocol for the motility assay was adapted from Barker *et al.* (58). The strains were cultured overnight in Tryptone Broth medium (TB, tryptone 10 g/l, sodium chloride 5 g/l) with shaking (200 rpm) at 33°C. A total of 2 μl of the overnight cultures were inoculated into semisolid TB agar with 0.3% (wt/vol) agar. After incubation for 24 h at 33°C, swarm diameters were measured. Photographs were taken with a Nikon DSLR camera.

## RESULTS

### Study design

tRNA modification levels can be assessed by different approaches (59,60), either globally, e.g. by LC-MS or similar techniques, or site-by-site, using deep sequencing-based methods of RNA modification analysis providing single nucleotide resolution. We previously extended the application of alkaline hydrolysis-based RiboMethSeq to analysis of *E. coli* tRNA 2′-O-methylations and demonstrated that the level of individual Nm residues can be precisely measured using this technique (48). Therefore, RiboMethSeq was used in this study to measure dynamic modulation of *E. coli* tRNA 2′-O-methylations under various stress conditions. Altogether, out of 20 potentially 2′-O-methylated sites in *E. coli* tRNAs (48), we were able to reliably detect and assess the 2′-O-methylation status of 17 of them (9 Gm18, 6 Um/Cm32 and 2 U*m/Gm34). Three residual Gm18 sites in rare isoacceptor tRNAs (tRNAIle(k^2^CAU), tRNAMet(ac^4^CAU) and tRNAGln(U*UG)) were excluded from analysis since sequencing coverage, particularly for the D-loop of these tRNAs, was insufficient for reliable quantitative assessment of the modification level.

Conditions of bacterial growth are known to affect the RNA(tRNA) modifications. Most prominent changes have been observed under stresses, such as nutritional deficiency, low or high temperature, reduced oxygen level or oxidative stress and the presence of various antibiotics at sub-lethal concentrations (18,27,34). In order to screen the maximum of potentially relevant stress conditions, we explored media composition (rich LB versus poor M9 medium), temperature (standard 37°C, versus 42°C and 20°C), starvation in PBS and reduced oxygen supply (hypoxia-like conditions) previously used for mycobacteria (34), as well as low concentration of antibiotics, producing bacteriostatic effects (sub-lethal antibiotic stress).

### Variations of tRNA 2′-O-methylation under stress conditions

**Figure 1.**
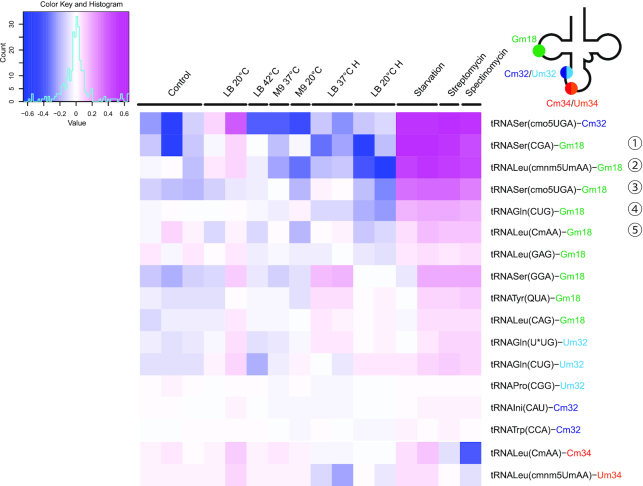
Initial screening of stress conditions for analysis of tRNA 2′-O-methylation dynamics in *Escherichia coli*. Heatmap represents MethScore values for measurable positions of 2′-O-methylation known in *E. coli* tRNAs. In order to assess biological variability, analysis was performed using independent biological replicates. Normalization of MethScore values was done as a difference to the average MethScore for all samples at a given position. Scale of MethScore variation is shown in the Color Key (top left). tRNA identity, anticodon and modified position are indicated on the right.

The first general screening of tRNA 2′-O-methylation levels at different growth conditions revealed that only few of the known 2′-O-methylated sites were constitutively modified in *E. coli* and did not show any substantial and/or reproducible variation under different stress conditions applied (Figure 1). These constitutively modified residues are mostly Um/Cm residues at position 32, but also some Gm18 methylations. Interestingly, and in some contrast, other tRNA 2′-O-methylated positions, including, in particular, a number of Gm18 sites, displayed substantial variations under stress, and specifically under starvation and stress created by sub-lethal antibiotic concentrations. The heatmap in Figure 1 shows variations of the MethScore level (row-normalized to average) site-by-site, and absolute numbers are given in the Supplementary Figure S2A and B. In most cases, a general increase of G18 2′-O-methylation was detected under stress, with only a few not-Gm18 cases (tRNALeu(CmAA)-Cm34), where a slight decrease was observed. Of note, other growth conditions, like poor medium (M9), low (20°C) or high growth temperature (42°C) and restricted oxygen supply (hypoxia-like conditions) had only a very mild effect on tRNA 2′-O-methylation, compared to starvation in PBS and antibiotics tested.

Since the most substantial variations of tRNA 2′-O-methylation levels were observed under starvation and sub-lethal antibiotic stress, we decided to focus our study on those conditions, and to also extend the list of antibiotics used. In addition to streptomycin and spectinomycin used for the initial screening, we also included chloramphenicol and gentamycin at sub-lethal concentrations. The choice of these antibiotics was based on their chemical structure and molecular mechanism of action: streptomycin and spectinomycin are aminoglycosides which bind to 16S rRNA and modulate ribosome fidelity and speed, altering codon-anticodon recognition and ribosome translocation, respectively. Chloramphenicol is a non-aminoglycoside antibiotic that inhibits ribosome peptidyltransferase activity by binding to 23S rRNA. Gentamycin, like streptomycin, binds 16S rRNA and affects mRNA translation fidelity and was included in the study since it is frequently used in clinics for treatment of beta-lactam-resistant bacterial infections. In order to reduce the biological variability, replicates in these series were derived from the same *E. coli* bacterial culture (experimental design is shown in Supplementary Figure S3).

**Figure 2.**
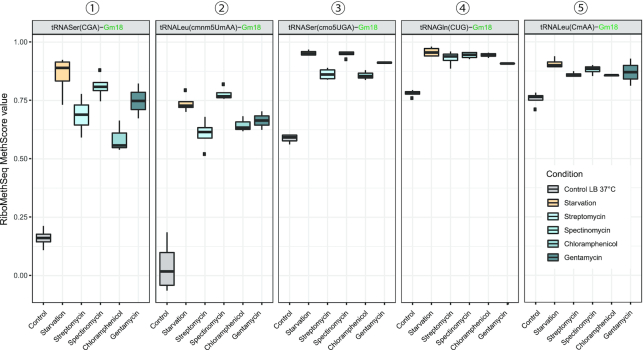
MethScore values for 5 most responsive positions in *Escherichia coli* tRNAs, observed under standard conditions (control LB 37°C) and under starvation in PBS (starvation) and sub-lethal antibiotic stress. The identity of analyzed tRNA position is shown on the top of each panel, identity of sample is indicated in color and shown on the bottom. Boxplot represents mean values (*n* = 2–4) as a bar, distance between (first and third quantile and a box), max/min values as whiskers and outliers as black dots.

Renewed RiboMethSeq analysis of tRNA 2′-O-methylation fully confirmed results identified in the initial screening (Figure 2 and Supplementary Figure S4A–D) and documented hypermethylation of certain ribose moieties as a common response toward all the various ribosome-acting antibiotics examined. Biological replicates provided very consistent results demonstrating that the level of Gm18 in 5 specific tRNAs strongly increased (Figure 2). In some instances, tRNA positions that appeared almost unmodified under standard growth conditions (e.g. Gm18 in tRNALeu(cmnm^5^UmAA) and tRNASer(CGA), see Figure 2, left and middle panels) became highly modified under stress. A similar trend was also observed for Cm32 in tRNASer(cmo^5^UGA), while levels of 2′-O-methylation for cmnm^5^Um34 and Cm34 were mildly decreased upon treatment with aminoglycosides and strongly reduced upon chloramphenicol sub-lethal stress (Supplementary Figure S4C and D).

The changes observed under stress were reversible and normal levels of 2′-O-methylation were restored when the aliquot of *E. coli* culture under stress was withdrawn and re-grown in a new LB batch at 37°C (Supplementary Figure S5).

In *E. coli*, 2′-O-methylated residues are found not only in tRNAs, but also in rRNA, where four sites are known (rRNA 16S-m^4^Cm1402, 23S-Cm2498/Gm2251/Um2552). To complete RiboMethSeq analysis, rRNA-mapped reads were recovered and used for calculation of MethScore at these positions. As shown in Supplementary Figure S6, rRNA 2′-O-methylation was not affected by stress conditions tested here, indicating that rRNA methylation is rather constitutive in *E. coli*.

### Validation of RiboMethSeq analysis by LC-MS

RiboMethSeq data clearly demonstrated that 2′-O-methylation signals observed in *E. coli* tRNAs were modulated under stress conditions tested. In order to confirm and validate these data by an orthogonal analytical technology, we performed LC-MS analysis of tRNA modification levels in the same samples. Nucleoside analysis of tRNA digest does not allow mapping to a given modification site, but rather provides a quantitative information on modulation of RNA modification content.

#### Total tRNA analysis

**Figure 3.**
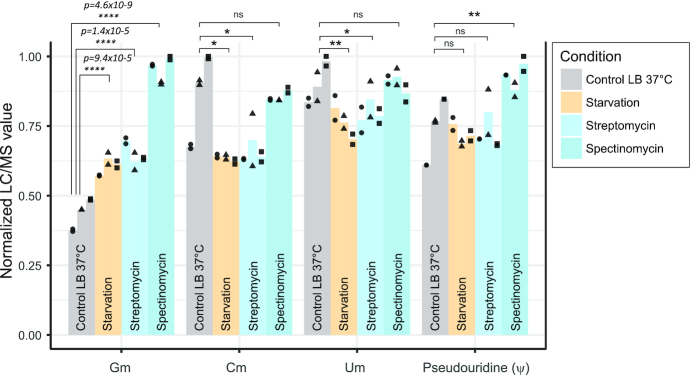
LC/MS analysis of global 2′-O-methylation content in total *Escherichia coli* tRNA fraction. Normalized LC/MS content of modified nucleoside is shown for 2′-O-guanosine (Gm), 2′-O-cytosine (Cm), 2′-O-uridine (Um) and pseudouridine (used as control here). Biological replicates (*n* = 3) and technical replicates (*n* = 2) are shown as color bars and black dots/triangles/squares, respectively. *: *P*-value < 0.05, **: *P*-value < 0.01, ****: *P*-value < 0.0001. Statistical values were calculated for technical and biological replicates using two-tailed *t*-test with unequal variance.

First, we performed global measurements of Gm, Cm, Um (Am residues are not present in *E. coli* tRNAs) and, for control purposes, pseudouridine content in total tRNA fractions obtained under control conditions (LB 37°C), as well as under starvation and antibiotic stress (streptomycin and spectinomycin). As shown in Figure 3, levels of Cm, Um were relatively stable or showed some decrease under stress and the pseudouridine level was only increased in spectinomycin condition. In contrast, global Gm level in tRNA fractions increased significantly under antibiotic stress and starvation. Of note, while this observed increase was in line with the RiboMethSeq results, it was observed for total tRNA and may vary for tRNA species taken individually.

#### Isolation and analysis of individual tRNAs

In order to attribute alterations of global Gm level in total tRNA fraction to individual variations by tRNA observed in RiboMethSeq, four individual tRNAs were isolated to highly homogeneous state by immobilized DNA-oligonucleotide affinity column (see Supplementary Figure S1). Purity of the preparations was confirmed by library preparation and sequencing (Supplementary Figure S7). Preparation of individual tRNAs was done for control condition LB 37°C, starvation in PBS as well as for three antibiotic stress conditions (streptomycin, spectinomycin and chloramphenicol).

**Figure 4.**
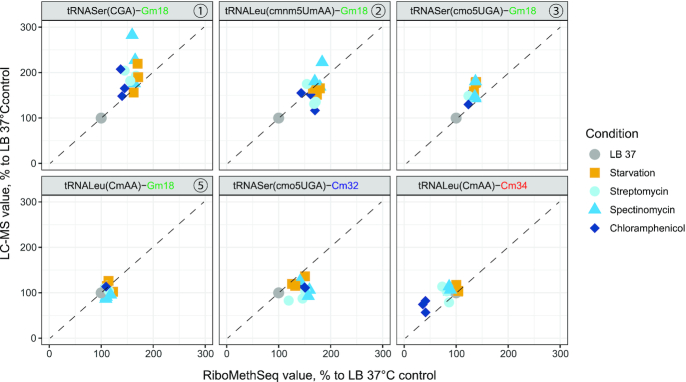
Scatter plots for correlation of LC-MS and RiboMethSeq data for 2′-O-methylation quantification. Variations are shown as a relative increase (or decrease) of 2′-O-methylation compared to control values observed for LB 37°C (control condition, in gray). Biological replicates (*n* = 3) are shown. The identity of measured tRNA position is indicated on the top of each panel, the nature of the stress is given in color and shape of the dot.

Figure 4 shows comparison of LC-MS measurements for Gm18, Cm32 and Cm34 levels in individual tRNAs with data obtained by RiboMethSeq analysis of the same samples.

Altogether, data showed very consistent measures of Gm18 tRNA content obtained by LC-MS and RiboMethSeq. In most cases, the dispersion among biological replicates data was lower for RiboMethSeq, compared to LC-MS measurements, even if the latter were performed in technical replicates. Only for Cm32 in tRNASer(cmo^5^UGA), LC-MS measurements showed invariability of the Cm32 content, while an increase was apparent in RiboMethSeq. This may be related to variations of other non-measured modifications in a close proximity of Cm32 (namely cmo^5^U34 at tRNA wobble position) and thus somehow affected the protection score for Cm32 calculated in RiboMethSeq approach. Apart from this discrepancy on cytidine ribose methylation, LC-MS and RiboMethSeq gave consistent results, with respect to the dynamic modulation of Gm18 in *E. coli* tRNAs under starvation and mild sub-lethal antibiotic stress.

### Gm18 tRNA modification does not affect tRNA translation efficiency

**Figure 5.**
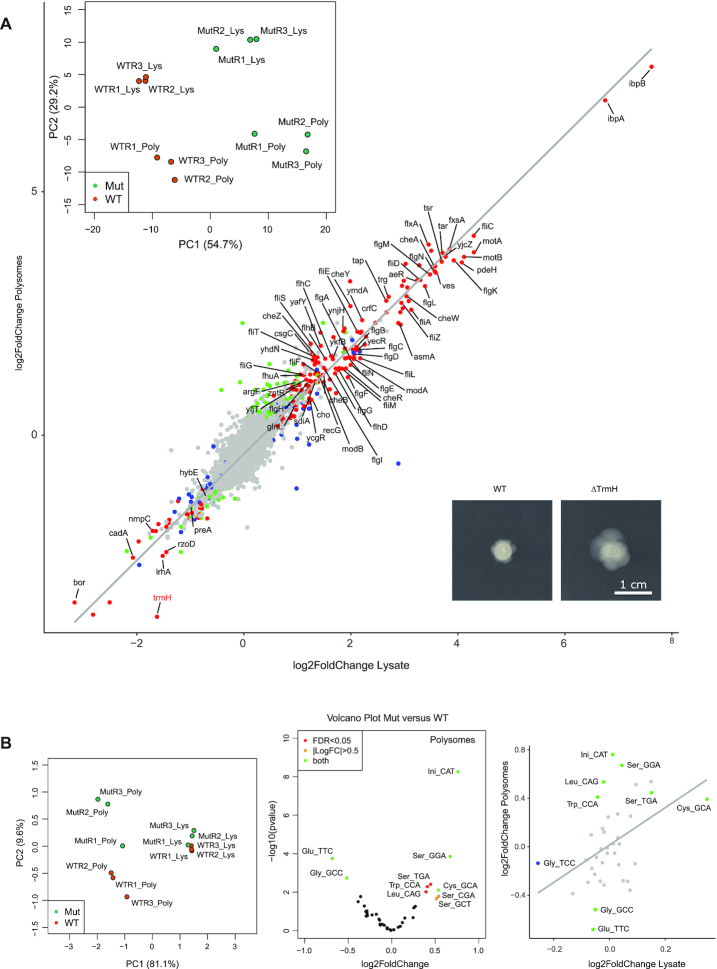
Comparative analysis of global mRNA/tRNA alterations and specific association to polysomes in WT and ΔTrmH *Escherichia coli* strains. (**A**) Correlation between polysomal association of *E. coli* mRNAs and their expression level in lysates for WT and ΔTrmH strains. Values are expressed in Log2FoldChange levels between WT and ΔTrmH strains. Points indicated show genes with altered level both in lysate and in polysomes (padj <0.0001). Genes affected only in lysate (or polysome fraction) are in green and blue, respectively. Identity of genes with altered expression and polysomal association is shown. Left top insert shows Principal Component Analysis (PCA) plot for mRNA expression and polysomal association in three biological replicates. Lower right insert illustrates increased swarming motility of ΔTrmH compared to WT cells (full data are shown in Supplementary Figure S8). (**B**) Altered polysomal association of tRNAs in ΔTrmH strain. Left panel shows PCA plot for biological replicates. Volcano plot (log10(padj value) versus log2FoldChange) for mutant ΔTrmH strain versus WT. Colors are attributed according to padj value and Log2FC (legend at top left corner). Scatter plot on the right panel (correlation of log2FoldChanges in Polysomes versus lysates) demonstrates globally identical level of tRNA expression (only tRNAGly(TCC) shows padj<0.05), but altered association with polysomal fraction (statistically significant points are indicated, padj<0.05).

In order to evaluate the importance of tRNA-Gm18 in bacterial translation, we performed a comparative analysis of tRNAs and mRNAs associated to polysomal fraction in WT and ΔTrmH *E. coli* strains. Both tRNAs and mRNAs were counted both in Lysate and Polysomal fractions, to determine alteration of tRNA/mRNA steady-state level, as well as association to polysomal fraction implicated in active translation. As shown in Figure 5A (insert), both total and polysomal fractions of *E. coli* mRNAs show consistent alteration upon deletion of TrmH. Proportion of polysome-associated mRNAs shows good a correlation with global mRNA expression in lysates (Figure 5A). Over 200 mRNAs were up- or downregulated upon TrmH deletion and a substantial proportion of deregulated genes belongs to pathways controlling chemiotaxis and motility. However, all over/under-expressed genes showed increase/decreased polysomal association, indicating that mRNA translation efficiency was not affected upon the absence of Gm18 modification in tRNA. Since the majority of mRNAs overexpressed in TrmH-deficient strain belongs to flagella/chemiotaxis/motility pathways, we compared motility of this *E. coli* strain compared to its WT counterpart. As shown in Figure 5A and Supplementary Figure S8, indeed, ΔTrmH strain showed a substantially increased motility on agarose plates.

Analysis of tRNA expression and association to polysomes demonstrated that tRNA expression (and stability) was unaffected when Gm18 was missing, while polysomal association was altered for a subset of tRNA species (Figure 5B). tRNAs Gly(GCC) and Glu(TTC) showed decreased association to translating ribosomes, while initiator tRNAfMet, as well as tRNAs Ser(GGA,TGA,CGA,GCT), Cys(GCA), Trp(CCA) and Leu(CAG) were substantially enriched. Of note, there was no correlation between the presence of Gm18 modification in *E. coli* tRNAs and their altered association with polysomal fraction upon TrmH deletion.

These results clearly indicate that tRNA-Gm18 has little impact on mRNA translation by the ribosome and tRNA stability, but rather selectively affects expression level of certain mRNAs as well as association of specific tRNAs to polysomal fraction, most likely as a part of adaptive response.

### Modulation of tRNA immunostimulation properties

One of the known biological functions of Gm18 modification in bacterial tRNA is its modulation of the human innate immune response, e.g. inhibition of IFN secretion by plasmacytoid dendritic cells. This effect is mainly mediated by antagonizing endosomal receptor TLR7 which recognizes a short nucleotide context (including Gm18 as a major identity determinant) in the tRNA D-loop (37–38,40–41). Since we observed substantial modulation of Gm18 *E. coli* tRNA content under stress conditions, we wondered if these changes were sufficient to also modulate TLR7-mediated immune response to such differentially modified tRNAs, even if Gm modified individual tRNAs only comprise a subfraction within total tRNA.

In order to measure immunomodulation, total tRNA fraction isolated under chloramphenicol stress was used to stimulate human pDCs followed by measurement of IFNα and TNFα release by ELISA. The level of IFNα release is known to be highly variable, depending on the source of donor PBMCs, thus measurements were made for 3 independent pDC preparations.

**Figure 6.**
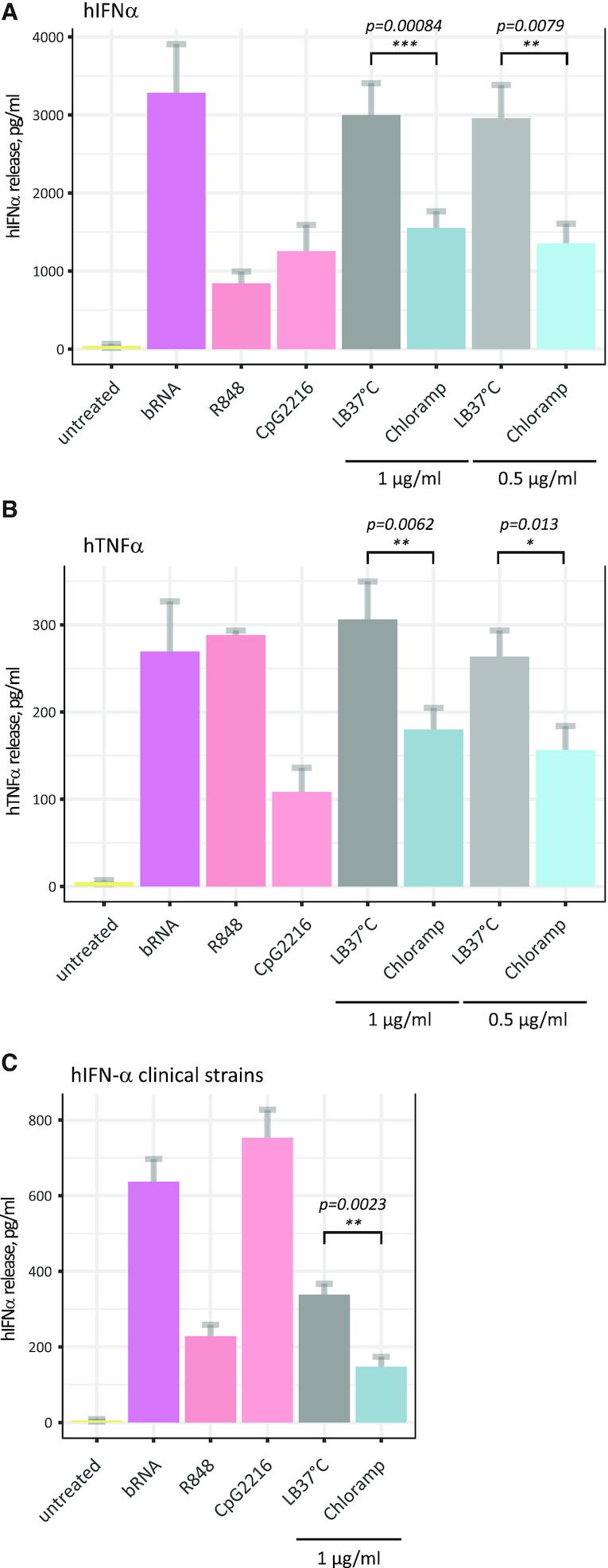
Immunostimulation properties of *Escherichia coli* total tRNA fraction measured using IFNα and TNFα release by human pDC and PBMCs. (**A** and**B**) Human pDCs from three healthy donors were stimulated with total tRNA from control conditions (LB 37°C) and from chloramphenicol treated *E. coli* lab strain culture at two different concentrations (1 μg/ml and 0.5 μg/ml). Total RNA from *Staphylococcus aureus* (bRNA), R848 and CpG2216 served as positive controls. The IFNα (**A**) and TNFα (**B**) release was measured for three biological replicates of tRNA fractions and in two technical replicates for ELISA measurements. (**C**) Human PBMCs from healthy donors were stimulated with total tRNA from three different clinical *E. coli* strains isolated from patient's urine samples at 1 μg/ml. The *E. coli* strains were cultured under control (LB 37°C) and stress conditions (chloramphenicol treated). Total RNA from *S. aureus* (bRNA), R848 and CpG 2216 served as positive control. The IFNα release was measured for three biological replicates of tRNA fractions and in two technical replicates for ELISA measurements. Error bars show standard error of the mean (s.e.m.).

As shown in Figure 6A and B and Supplementary Figure S9, IFNα and TNFα release by pDCs treated with total bacterial tRNAs obtained under chloramphenicol stress (and thus having high Gm content) was significantly reduced compared to treatment with the same concentrations of total tRNA preparation obtained from control bacterial culture (LB 37°C). It should be taken into account, that only a proportion of Gm18-containing tRNAs shows differential methylation, while modification level of other species remains relatively stable.

In order to verify if the increased level of methylation and thus decreased immunostimulation properties of total tRNA observed for a laboratory *E. coli* strain can be also reproduced in clinical settings, clinical *E. coli* isolates obtained from patients were also subjected to stress. Total tRNA fractions were used to determine both immunostimulation and methylation pattern. As shown in Figure 6C, total tRNA fractions extracted from clinical strains follow the same trend that was observed for the laboratory *E. coli* strain: tRNAs from *E. coli* cultured under stress conditions are significantly less stimulatory compared to tRNAs from control conditions. Furthermore, according to the RiboMethSeq data (Supplementary Figure S10), more Gm methylation in tRNA extracted from stressed bacteria was observed, correlating again very clearly with a decrease in immunostimulation.

We conclude, that this bacterial adaptation of tRNA modification to *antibiotic stress mediates a biological effect not related to bacterial translation*, namely a modulation of immunostimulatory properties.

## DISCUSSION

Modulation and interdependence among RNA modifications (mostly in tRNA, but now also in mRNA) in response to abnormal (stress) physiological states of the cell are well established (15,28,30). Not only is the presence of various RNA modifications known to be required for adequate stress response (21,61–63), but also have more and more cases of their modulation under stress conditions been reported (14,27,64–67). However, in the majority of cases, such changes in RNA modification profile were appreciated only by global analysis of modified nucleoside content in total RNA fraction, or after partial fractionation e.g. isolation of total tRNA (13,17–18,22,68). These observed global differences may be also related to modulation of the tRNA pool composition (69,70), potentially affecting global modified nucleoside content (71,72). Such adaptive regulation of the tRNA pool was observed e.g. for lactic bacteria *Lactococcus lactis* (73). In a *Caenorhabditis elegans* animal starvation model, a dynamic regulation of m^1^A, m^5^C, m^7^G, m^5^U in large RNAs, as well as ac^4^C and mcm^5^s^2^U in tRNAs was revealed by high-resolution LC-MS analysis (68).

In contrast to previously reported global evaluations of RNA modification content in tRNAs (74,75), a detailed analysis of 2′-O-methylation status by tRNA species and by position was performed in our study. High-throughput profiling of tRNA 2′-O-methylation by RiboMethSeq allowed to measure modulation of tRNA modification under various stress conditions. We found that temperature and nature of nutrients in the growth media have only minor effects on tRNA ribose methylations’ status, while starvation (PBS) and antibiotic stress lead, somewhat surprisingly, to a clearly significant increase. Modulation of tRNA 2′-O-methylation was not simply linked to a slow growth (and thus longer contact time with tRNA substrate and the enzyme modifying these substrates), since no visible increase in methylation was observed for ‘slow growth’ cultures in M9 minimal media at 20°C. Moreover, changes in 2′-O-methylation were observed only for a subset of possible tRNA substrates.

This finding is striking, since the effect of modifications in the structural core of the tRNA, such as Gm18, are known to increase tRNA stability (2–6,30,76), and thus the observed effect would be expected to enhance translation. This is in contrast with the typical stress response, namely a decrease or even a shutdown of translation (75,77–80). Polysome profiling of WT and ΔTrmH strains clearly points out that only mRNA steady state level is affected in the mutant, but not mRNA association to polysomes (translation). In contrast, tRNA steady-state level is not at all affected in the mutant, only preferential association of specific tRNAs to polysomes was observed. Hence a plausible hypothesis may be that increase of Gm18 content mostly impacts molecular events outside bacterial translation. In support of such hypothesis, we here documented a molecular mechanism that translates stress factors such as limited nutrient supply (starvation) or sub-lethal antibiotic stress, into molecular antagonists that inhibit RNA immunostimulation of the human innate immune system.

A logical next step, namely to address these findings *in vivo*, currently suffers from the lack of a suitable *in vivo* infection model. Given that the RNA/TLR7 axis is the only one in a large arsenal of PAMP/PRR systems (43,81) in the innate immune system, it stands to reason, that the contribution of tRNA to immunostimulation by living bacteria (46,82–84) might constitute an effect that is difficult to tease out *in vivo*. Clearly, while an assessment of the overall impact of bacterial Gm18 modulation under stress on infection remains to be characterized, we have here provide a causal chain along with a molecular mechanism that links exposure to antibiotics to a reduction of interferon emission by cells of the human innate immune system.

## DATA AVAILABILITY

*E. coli* polysome profiling data are deposited to the European Nucleotide Archive, accession number PRJEB41080

## Supplementary Material

gkaa1123_Supplemental_File
