# Supplementary material for: Bacterial tRNA 2′-O-methylation is dynamically regulated under stress conditions and modulates innate immune response
Source: Nucleic Acids Res. 2020 Dec 4;48(22):12833–44. doi: 10.1093/nar/gkaa1123 (PMC7736821; doi:10.1093/nar/gkaa1123)
Supplement: gkaa1123_Supplemental_File [file gkaa1123_supplemental_file.docx]

# Supplement

# Material and Methods

## Bacterial strain and stress conditions used

LB 20°C and LB 42°C were grown in LB medium to mid-log phase at 20°C and 42°C respectively at 190 rpm. M9 37°C and M9 20°C were grown in the minimal medium M9 (33.7 mM Na_2_HPO_4_, 22.0 mM KH_2_PO_4_, 8.55 mM NaCl, 9.35 mM NH_4_Cl, 0.4% glucose, 1 mM MgSO_4_, 2.3 mM CaCl_2_,1 µg/mL thiamine) to mid-log phase at 37°C and 20°C, respectively at 190 rpm. Hypoxia like conditions: LB 37°C Hypoxia and LB 20°C Hypoxia were grown in 50 mL Falcon tubes full of LB medium and incubated without shaking during 24h at 37°C and 20°C, respectively. For PBS starvation, a control culture (mid-log phase) was centrifuged for 5 min at 3,500xg and washed with 5 mL of 1X Phosphate-buffered saline (PBS) (8.1 mM Na_2_HPO_4_, 1.47 mM KH_2_PO_4_,137 mM NaCl, 2.7 mM KCl, pH 7.2-7.4). After another centrifugation step of 5 min at 3,500xg, cell pellet was resuspended in PBS (the same volume as initial LB medium) and incubated for 24h at 37°C, 190 rpm. For sub-lethal antibiotic stress conditions, a control culture (mid-log phase) was supplemented with the following concentrations of antibiotics: spectinomycin at 10 µg/mL, streptomycin, chloramphenicol and gentamycin at 5 µg/mL and then incubated for 24h at 37°C and 190 rpm.

## tRNA extraction

Protocol for bacteria tRNA extraction (1). For all conditions, cells were harvested in 50 mL Falcon tubes by centrifugation at 3,500g for 5 min. Cell pellet was washed with 5 mL of 1x PBS. After another centrifugation step of 5 min at 3,500g, pellet was directly used for direct total tRNA isolation by TRIzol^TM^ reagent (Thermo Fisher Scientific) according to the manufacturer’s instructions. Briefly, pellet was resuspended in 3 mL of TRIzol™ and separated in 3x1.5 mL microcentrifuge tubes. Samples were vortexed and incubated for 5 min at room temperature (RT) and centrifuged for 5 min at 12,000xg. Supernatant was transferred in a new 1.5 mL microcentrifuge tube and 200 µL of chloroform was added. After vortexing, samples were centrifuged for 15 min at full speed 12,000-15,000xg. The aqueous phase was transferred to a new 1.5 ml microcentrifuge tube. RNAs were then precipitated with 500 µL of isopropanol and 1 µL of Glycoblue™ (15 µg). Tubes were cooled down for at least 30 min at -80°C and centrifuged for 30 min at full speed (12,000-15,000xg) at 4°C. RNA pellet was washed with 500 µL of 75% ethanol and centrifuged for 10 min at full speed (12,000-15,000xg) at 4°C. tRNA pellet was resuspended in milliQ water and stored at -80°C.

## Isolation of individual tRNA species

Isolation of individual tRNA was done using GE Healthcare HiTrap™ NHS-Activated HP Columns (1 mL). Synthetic DNA Oligonucleotides (targeting four tRNAs of interest: Leu(cmnm^5^UmAA), Ser(cmo^5^UGA), Ser(CGA) and Leu(CmAA)), described in the Supplementary Table S1) were covalently attached to the column via their primary amine at the 5’-end. Coupling was done using the following protocol (2): column was flushed with 6 mL of cold 1 mM HCl and NH2-DNA oligonucleotide in coupling buffer (0.2 M NaHCO_3_, pH 8.3, 0.5 M NaCl) was introduced into the column and incubated for 2 h at RT. The column was washed with 6 mL of buffer A (0.5 M ethanolamine, pH 8.3, 0.5 M NaCl) and buffer B (0.1 M sodium acetate, pH 4, 0.5 M NaCl). A second wash of buffer A was performed with an incubation time of 30 min. Then, the column was washed with 6 mL of buffer B, 6 mL of buffer A and again 6 mL of buffer B. After these steps the column is ready for isolation of individual tRNA or can be stored before use in a neutral phosphate buffer (50 mM Na_2_HPO_4_ pH 7.1, 0.1% NaN_3_).

For isolation of individual tRNAs, the column was first equilibrated for few minutes at 65°C. In parallel, total tRNA sample was prepared. 1 mg of RNA was denatured by heating for 2 min at 70°C in 1 mL of tRNA binding buffer (30 mM Hepes-KOH, pH 7.4, 1.2 M NaCl) and directly put on ice. Sample was transferred in a 50 mL Falcon tube containing 14 mL of tRNA binding buffer and was connected to the HiTrap column and placed into an oven at 65°C. Total tRNA sample was recirculated at 65°C for at least 1 hour using a peristaltic pump. After binding step, the column was washed with 10-15 mL of wash buffer (10 mM Hepes-KOH, pH 7.4, 100 mM NaCl) at 55°C. Individual tRNA bound to the column was eluted with 7 mL of elution buffer (1 mM Hepes-KOH, pH 7.4, 1 mM EDTA) by placing the column for 5 min into a preheated water bath at 75°C. Eluted fractions were precipitated by isopropanol (1/1 volume). Samples were resuspended in 20 µL milliQ water and loaded on a 10% urea denaturing gel. Band of interests were cut out, frozen for 30 min in -20°C and were eluted with 200 µL of 0.5 M NH_4_OAc. After shaking overnight at 750 rpm at RT, the gel suspension was filtered through centrifugal filters (NanoSeps 0.45 µm, VWR) and the resulting filtrate was ethanol precipitated.

Table S1: Sequence of oligonucleotides used for individual tRNA isolation

| **Targeted tRNA** | **Position of interest** | **Modifica-tion** | **Length** | **Sequence (5’->3’)** |
| --- | --- | --- | --- | --- |
| Leu cmnm^5^UmAA | Gm18 | 5' hexyl linked primary amine | 40 nt | TGGTACCCGGAGCGGGACTTGAACCCGCACAGCGCGAACG |
| Ser cmo^5^UGA | Gm18  Cm32 |  |  | TGGCGGAAGCGCAGAGATTCGAACTCTGGAACCCTTTCGG |
| Leu CmAA | Gm18  Cm34 |  |  | TGGTGCCGAAGGCCGGACTCGAACCGGCACGTATTTCTAC |
| Ser CGA | Gm18 |  |  | TGGCGGAGAGAGGGGGATTTGAACCCCCGGTAGAGTTGCC |

## RiboMethSeq analysis of 2’-O-methylations in tRNAs

### Alkaline hydrolysis

Alkaline hydrolysis of total *E.coli* tRNA was performed in bicarbonate buffer at 50 mM pH 9.2 for 10 min. The reaction was stopped by ethanol precipitation using 0.3 M sodium acetate, pH 5.2 and 1 µL of glycoblue™. After centrifugation, the pellet was washed with 80% ethanol and resuspended in nuclease-free water.

### Library preparation

RNA fragments without any gel-purification step were directly 3’-end dephosphorylated using 5 U of Antarctic phosphatase (New England Biolabs) for 30 min at 37°C. After the inactivation of the phosphatase for 5 min at 70°C, RNA fragments were phosphorylated at the 5’-end using T4 polynucleotide kinase (PNK) and 1 mM ATP for 1 h at 37°C. End-repaired RNA fragments were then purified using the RNeasy MinElute Cleanup kit (QIAGEN) according to the manufacturer’s recommendations, except that the volume of 96% ethanol was adjusted for RNA binding. Elution was performed in 10 µL of nuclease-free water. RNA fragments were converted to libraries using the NEBNext Small RNA Library kit (New England Biolabs) following the manufacturer’s instructions. DNA library quality was assessed using a High Sensitivity DNA chip on a Bioanalyzer 2100. Library quantification was done using a fluorometer (Qubit 2.0 fluorometer, Invitrogen). The library mix for sequencing was adjusted to obtain ~15–20 millions of raw reads for each library.

### Sequencing

Library sequencing was performed using the Illumina HiSeq 1000 sequencer in a single read mode for 50 nt (SR50). Primary analysis of sequencing quality was done with Illumina RTA 2.12 software, to insure a >Q30 quality score for >95% of the obtained sequences. Following the SR50 sequencing run, demultiplexing was done with Illumina BclToFastq v2.4; reads not passing the quality filter were removed.

### Data Analysis

Raw reads after demultiplexing were trimmed to remove the sequence of the Illumina 3’ adapter. Trimming was performed with Trimmomatic v0.32 (3) with the following parameters: -phred33 /adapters/TruSeq3-SE.fa:2:30:10 LEADING:30 TRAILING:30 SLIDINGWINDOW:4:15 MINLEN:8 AVGQUAL:30. Only trimmed and adapter-free reads of <35 nt were taken for alignment. Alignment to the reference tDNA sequences was performed with Bowtie2 ver2.2.4 (4) in end-to-end mode using the following parameter set: –no-1mm-upfront -D 15 -R 2 -N 0 -L 10 -i S,1,1.15. Uniquely-mapped reads were extracted from the *.sam file by RNA ID and converted to *.bed format using bedtools v2.25.0 (5). Positional counting of the 5’-and 3’-ends of each read was performed with the awk Unix command. Further treatment steps were done in the open-source R environment (v3.5.1) (The R Foundation. Available online: https://www.r-project.org/foundation/). In brief, 5’-end and 3’-end counts were merged together by RNA position and used for the calculation of ScoreMean (derived from the ScoreMax described previously), as well as Scores A and B (6) and MethScore (7). Scores were calculated for 2 neighboring nucleotides instead of 6 in the standard RiboMethSeq procedure (8). Profiles of RNA cleavage at selected positions were extracted and visually inspected.

## Digestion of tRNA samples to nucleosides and LC-MS(MS) analysis

Total tRNA or individual tRNA samples (150-300 ng of RNA) were digested into nucleosides by overnight incubation at 37°C in 25 mM NH_4_OAc (pH 7.5) by the following enzyme cocktail: 10 U of Benzonase (Sigma Aldrich), 2 U of thermosensitive Alkaline Phosphatase (Thermo Fisher Scientific), 0.6 U of nuclease P1 (Sigma Aldrich), 0.2 U of snake venom phosphodiesterase (Worthington Biochemical Corporation), 200 ng of Pentostatin (Sigma Aldrich) and 500 ng of tetrahydrouridine (Merck) which are adenosine and cytidine deaminase inhibitors, respectively.

Digested RNA (equivalent of 40 ng of initial sample) were mixed with 10 ng of ^13^C *S. cerevisiae* total tRNA digest: internal standard and used for LC-MS/MS measurement in order to quantify the level of 2’-O-methylated nucleosides in the samples.

Analysis on the nucleoside compositions was performed with an Agilent 1260 Infinity system with binary pump in combination with an Agilent 6460 triple quadrupole mass spectrometer with an electrospray ion source as described (9–11).

A C18 reverse phase HPLC column (Synergi™ 4 µm Fusion-RP 80 Å, LC Column 250 x 2 mm, Phenomenex) was used at a temperature of 35°C. A rising gradient of 100% LC-MS grade acetonitrile and a 5 mM NH_4_OAc buffer (pH 5.3, adjusted with acetic acid) was applied with a flow rate at 0.35 mL/min: 0 min/0% acetonitrile, 10 min/ 5% acetonitrile, 20 min/40% acetonitrile, 23 min/0% acetonitrile, 30 min/0% acetonitrile.

The four main nucleosides were detected photometrically at 254 nm whereas detection of the RNA modifications was conducted via triple quadrupole in the positive ion mode. The parameters of the electrospray ion source (Agilent Jet Stream electrospray ion source) were: gas temperature 350°C, gas flow 8 L/min, nebulizer pressure 50 psi, sheath gas temperature 350°C, sheath gas flow 12 L/min, capillary voltage 3,000 V, nozzle voltage 500 V.

For quantification experiments the dMRM mode of the LC-MS/MS was used. Following are the triple quadrupole settings required for adjustment in the Supplementary Table S2.

Table S2 Triple quadrupole settings

| Modification | Precursor ion (m/z) | Product ion (m/z) | Retention time (min) | Retention time window (min) | Fragmentor voltage (V) | Collision energy (V) | Cell accelerator voltage (V) |
| --- | --- | --- | --- | --- | --- | --- | --- |
| Cm | 258 | 112.1 | 9.3 | 3 | 60 | 9 | 2 |
| Cm ^13^C | 269 | 116.1 | 9.3 | 3 | 60 | 9 | 2 |
| Gm | 298 | 152 | 12.4 | 3 | 72 | 5 | 2 |
| Gm ^13^C | 309 | 157 | 12.4 | 3 | 72 | 5 | 2 |
| Um | 259 | 113 | 11.3 | 2 | 66 | 5 | 2 |
| Um ^13^C | 269 | 117 | 11.3 | 2 | 66 | 5 | 2 |
| Pseudouridine | 245 | 209 | 3.8 | 2 | 81 | 5 | 2 |
| Pseudouridine ^13^C | 254 | 218 | 3.8 | 2 | 81 | 5 | 2 |

Subsequent analysis of the peaks with predefined m/z values was performed with the Agilent MassHunter Software, Qualitative Analysis (V. 5.0.519.0). Extraction of ion chromatograms allowed integration of respective peaks, providing values for the area under the curve. In order to normalize these values, the following calculation was performed: x= ((dMRM(^12^C)/dMRM(^13^C)/(UV(A)-UV(A)_internal standard_)). dMRM (^12^C) or (^13^C) correspond to values for the area under the curve for ^12^C (Sample) and ^13^C molecules (internal standard), UV (A) and UV(A)_internal standard_ corresponds to the peak area of Adenosine in the UV chromatogram of the analyzed sample and alone internal standard sample, respectively. For comparison between control and stress conditions, control values were reported to 100 % and stress values were subjected to this calculation: y=(x(stress)/(x(associated control)-1))*100 where x corresponds to previously calculated values.

## Supplementary references

1. Galvanin,A., Ayadi,L., Helm,M., Motorin,Y. and Marchand,V. (2019) Mapping and Quantification of tRNA 2’-O-Methylation by RiboMethSeq. *Methods Mol. Biol.*, **1870**, 273–295.

2. Drino,A., Oberbauer,V., Troger,C., Janisiw,E., Anrather,D., Hartl,M., Kaiser,S., Kellner,S. and Schaefer,M.R. (2020) Production and purification of endogenously modified tRNA-derived small RNAs. *RNA Biol*, 10.1080/15476286.2020.1733798.

3. Bolger,A.M., Lohse,M. and Usadel,B. (2014) Trimmomatic: a flexible trimmer for Illumina sequence data. *Bioinformatics*, **30**, 2114–2120.

4. Langmead,B., Trapnell,C., Pop,M. and Salzberg,S.L. (2009) Ultrafast and memory-efficient alignment of short DNA sequences to the human genome. *Genome Biol.*, **10**, R25.

5. Quinlan,A.R. (2014) BEDTools: The Swiss-Army Tool for Genome Feature Analysis. *Curr Protoc Bioinformatics*, **47**, 11.12.1-34.

6. Birkedal,U., Christensen-Dalsgaard,M., Krogh,N., Sabarinathan,R., Gorodkin,J. and Nielsen,H. (2015) Profiling of ribose methylations in RNA by high-throughput sequencing. *Angew. Chem. Int. Ed. Engl.*, **54**, 451–455.

7. Marchand,V., Blanloeil-Oillo,F., Helm,M. and Motorin,Y. (2016) Illumina-based RiboMethSeq approach for mapping of 2’-O-Me residues in RNA. *Nucleic Acids Res.*, **44**, e135.

8. Pichot,F., Marchand,V., Ayadi,L., Bourguignon-Igel,V., Helm,M. and Motorin,Y. (2020) Holistic Optimization of Bioinformatic Analysis Pipeline for Detection and Quantification of 2’-O-Methylations in RNA by RiboMethSeq. *Front Genet*, **11**, 38.

9. Kellner,S., Ochel,A., Thüring,K., Spenkuch,F., Neumann,J., Sharma,S., Entian,K.-D., Schneider,D. and Helm,M. (2014) Absolute and relative quantification of RNA modifications via biosynthetic isotopomers. *Nucleic Acids Res.*, **42**, e142.

10. Thüring,K., Schmid,K., Keller,P. and Helm,M. (2016) Analysis of RNA modifications by liquid chromatography-tandem mass spectrometry. *Methods*, **107**, 48–56.

11. Thüring,K., Schmid,K., Keller,P. and Helm,M. (2017) LC-MS Analysis of Methylated RNA. *Methods Mol. Biol.*, **1562**, 3–18.


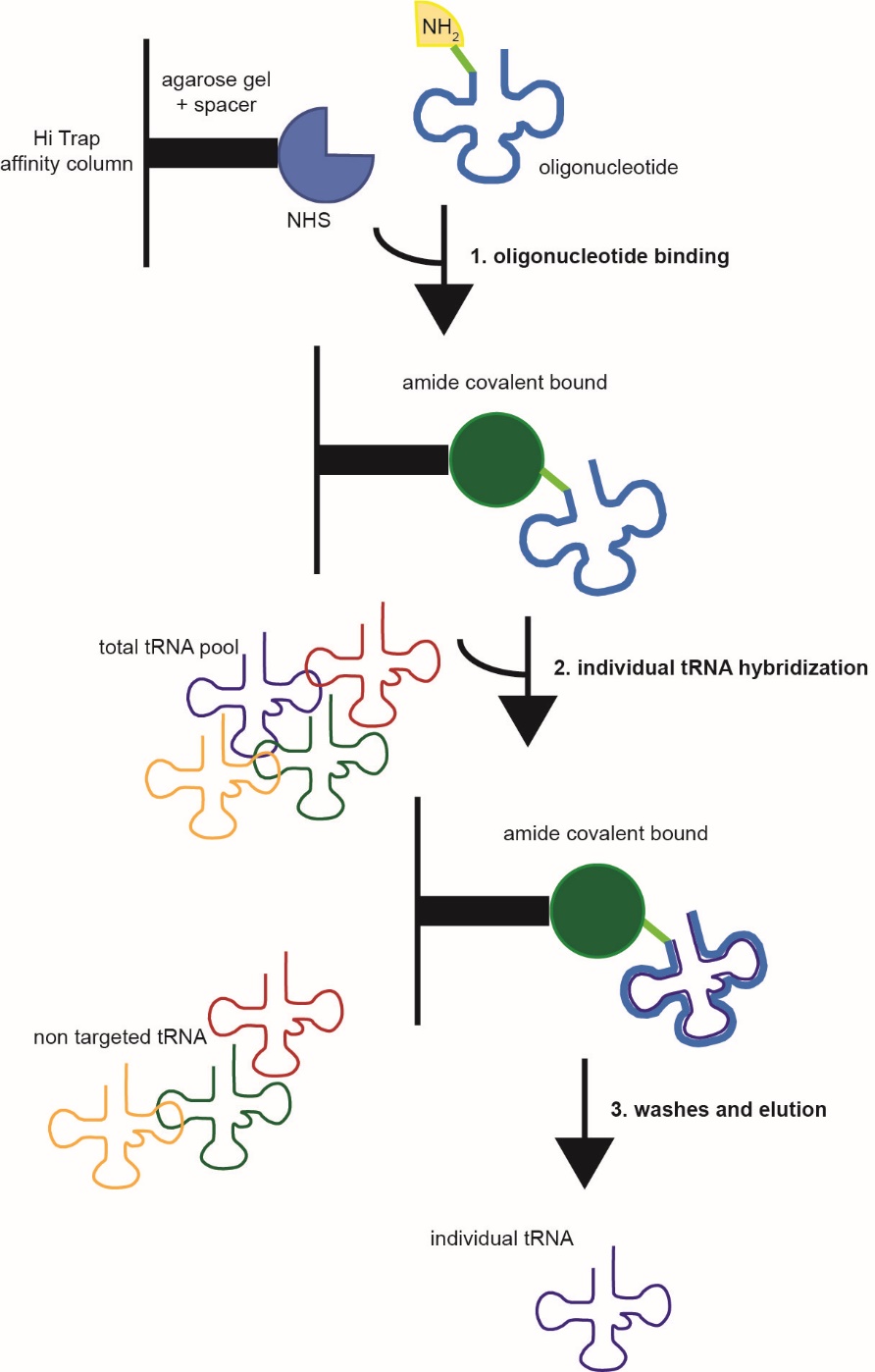


Supp Figure S1. Overview of individual tRNA isolation by DNA oligonucleotide affinity chromatography. Synthetic NH2-DNA oligonucleotide is covalently linked to activated NHS-Sepharose column (HiTrap 1 ml). Immobilized DNA oligonucleotide is used for hybridization to specific tRNA sequence in the total tRNA fraction. Elution was done with buffer at low ionic strength at 75°C.


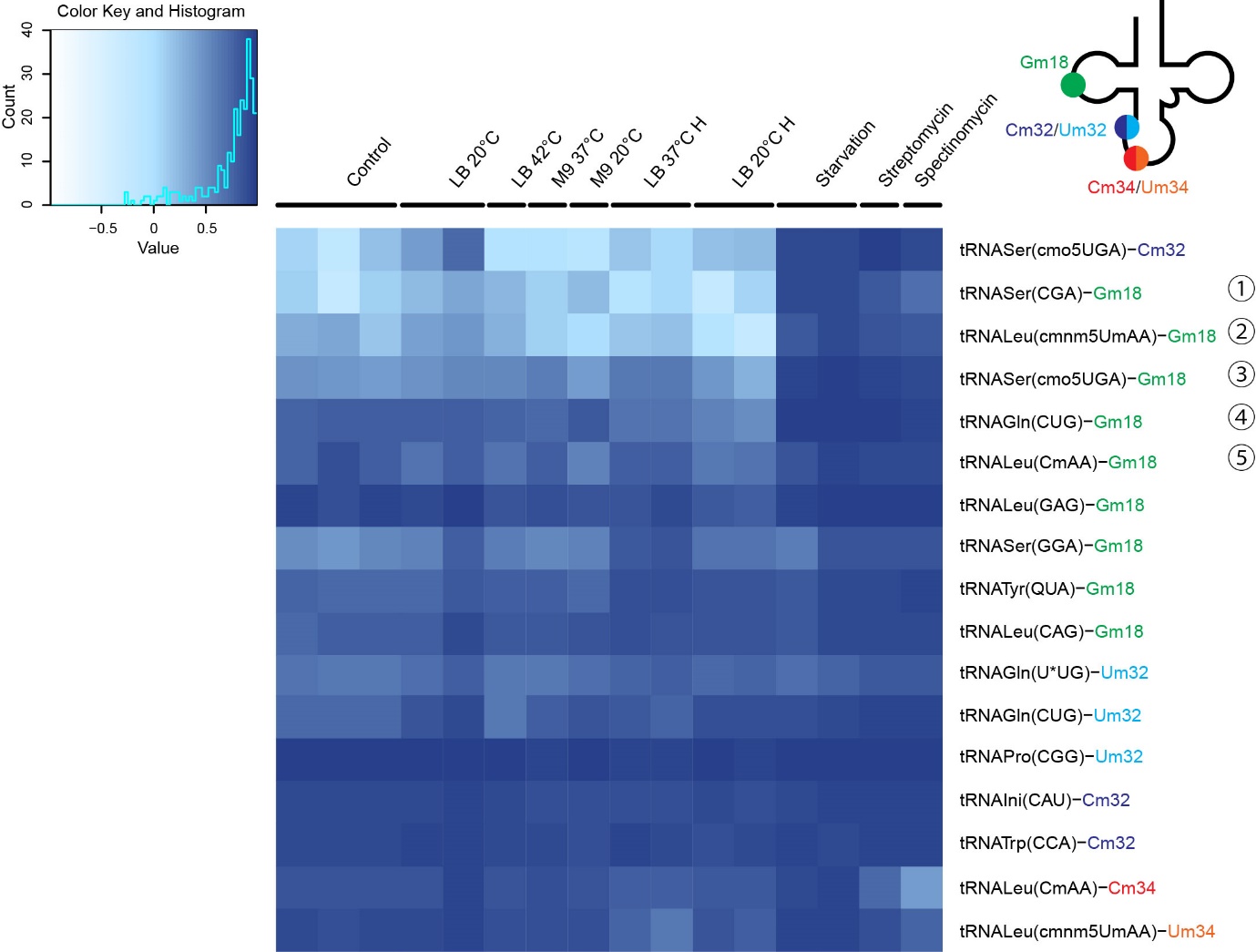


Supp Figure S2A. Initial screening of stress conditions for analysis of *E. coli* 2’-O-methylation dynamics. Panel A shows heatmap for absolute values of MethScore by position in tRNA (shown on the right) under different stress conditions and control (shown at the bottom). Scale of values is shown in the Color key (top left).


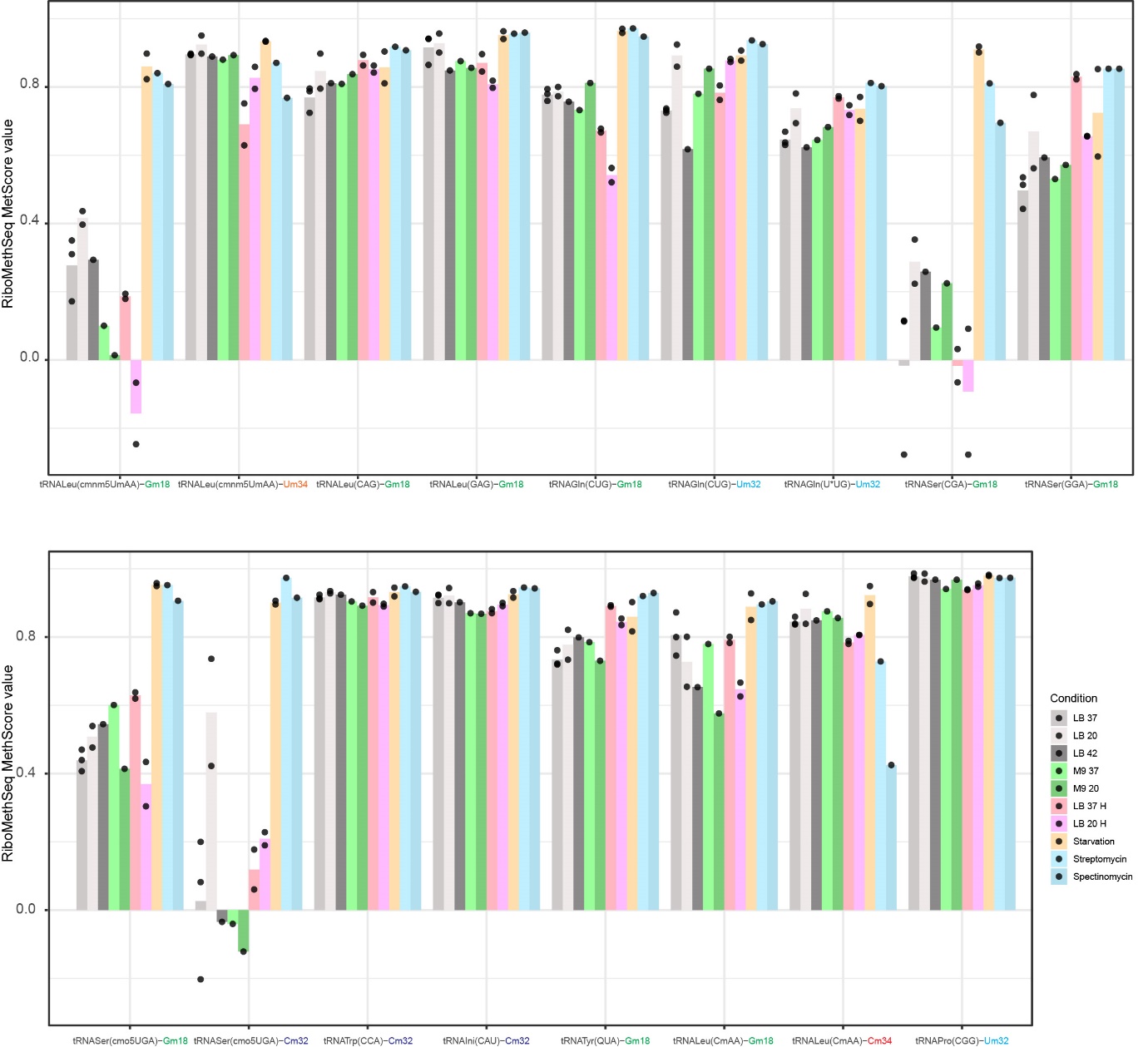


Supp Figure S2B. Initial screening of stress conditions for analysis of *E. coli* 2’-O-methylation dynamics. Panel B - barplot representation of the initial screening data, showing individual biological replicates for all tested conditions. Color bars corresponds to the average values; black dots show measurements for biological replicates.


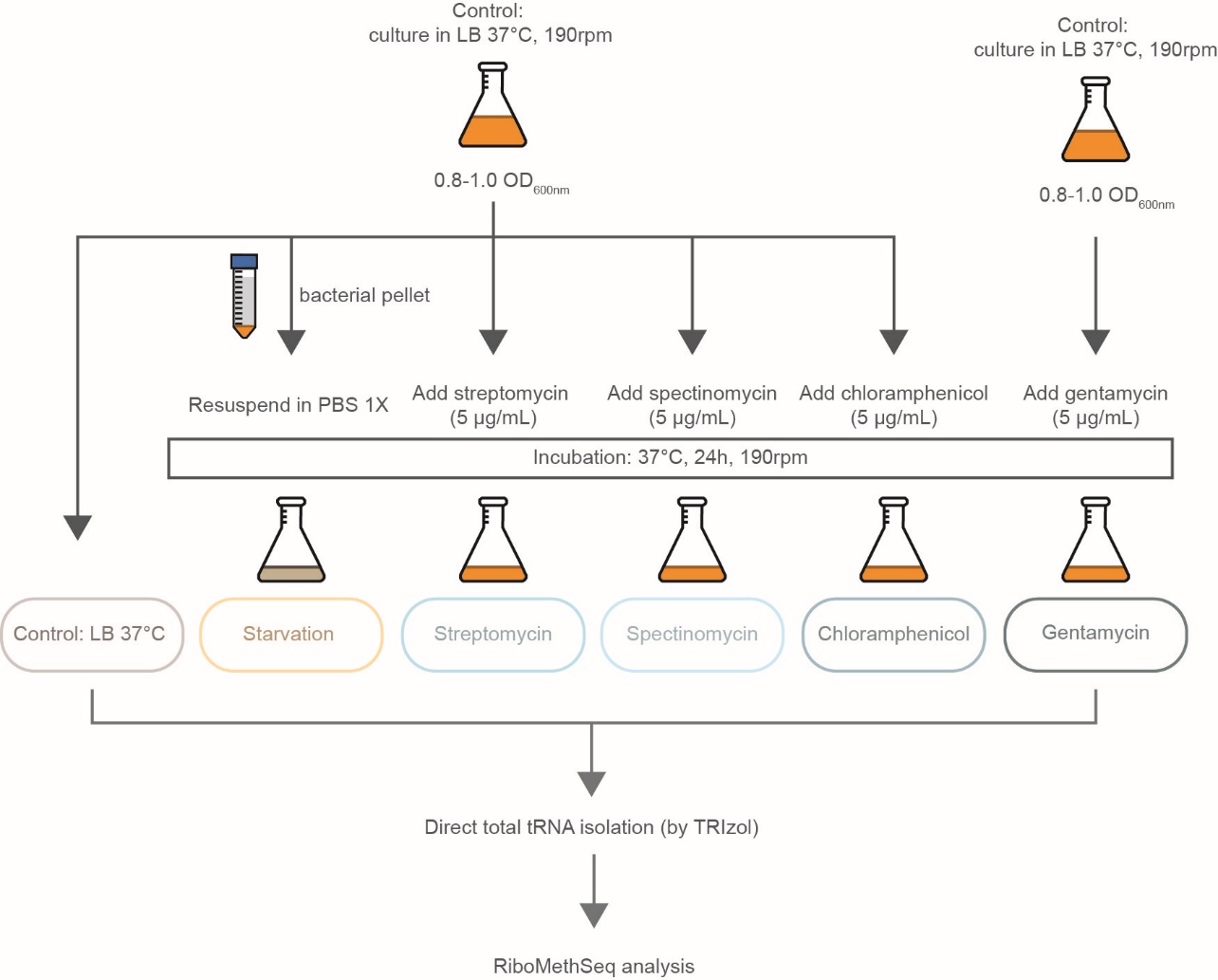


Supp Figure S3. Experimental design for selected stress conditions. Samples for starvation in PBS and three antibiotic stress (streptomycin, spectinomycin and chloramphenicol) were taken from the same culture, only gentamycin stress was measured separately.


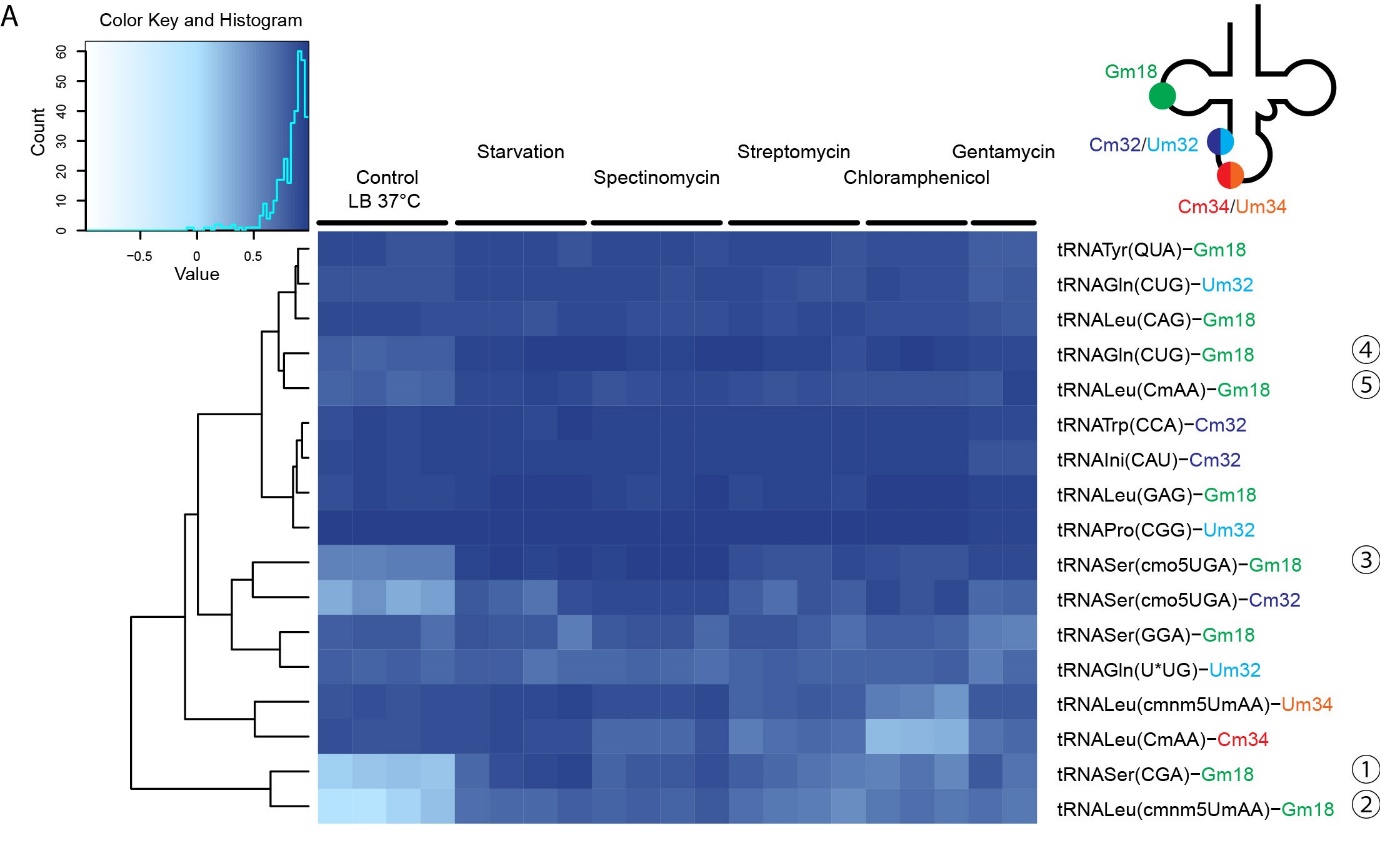


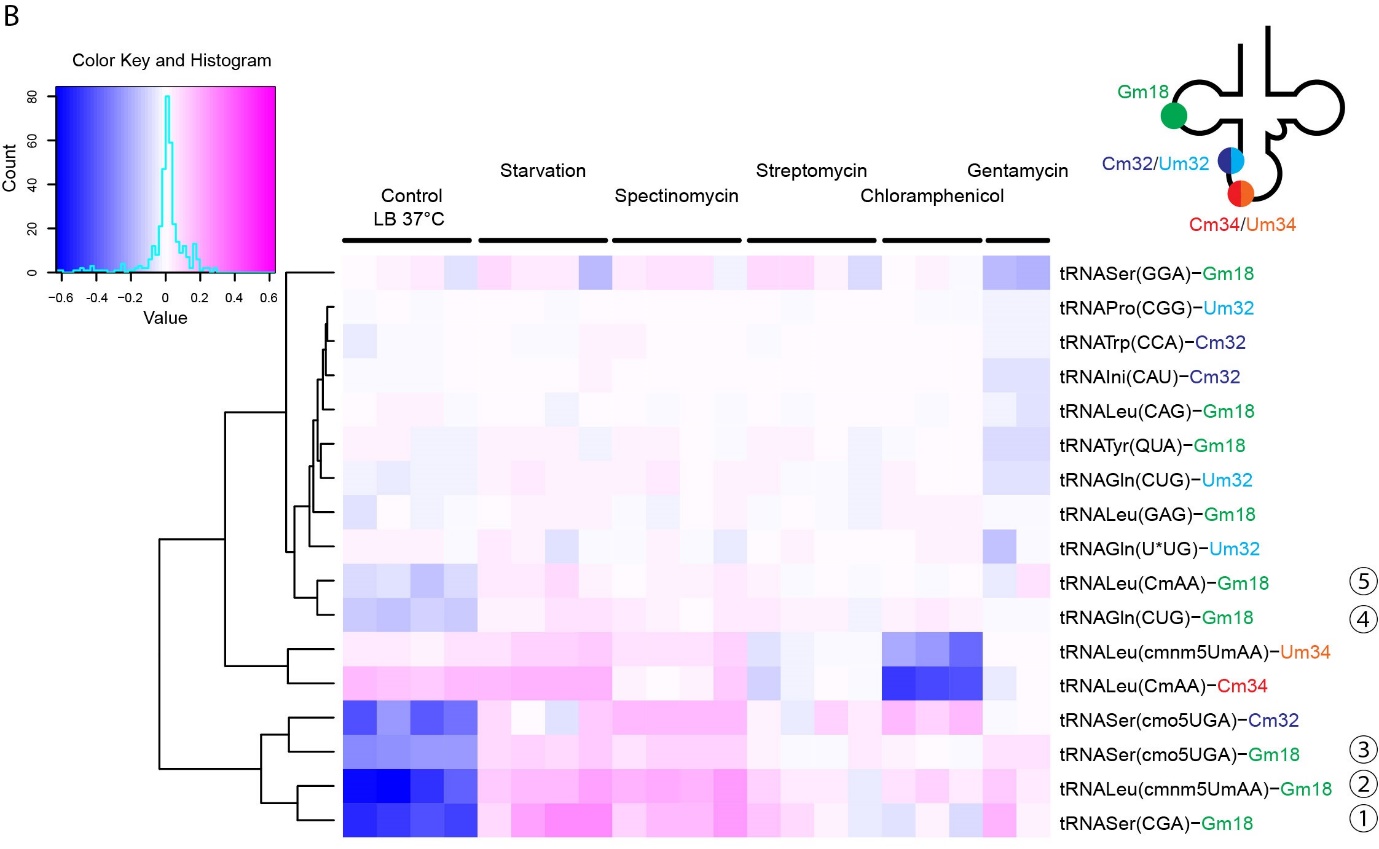


Supp Figure S4AB. RiboMethSeq analysis of 2’-O-methylation for selected stress conditions (2 to 4 biological replicates). Panel A (top) - heatmap for absolute values of MethScore by position in tRNA (shown on the right) in different stress conditions and control (shown at the bottom). Scale of values is shown in the Color key (top left). Panel B (bottom) - the same values on MethScore are given normalized to the average value by position. Legend is the same as for Panel A.


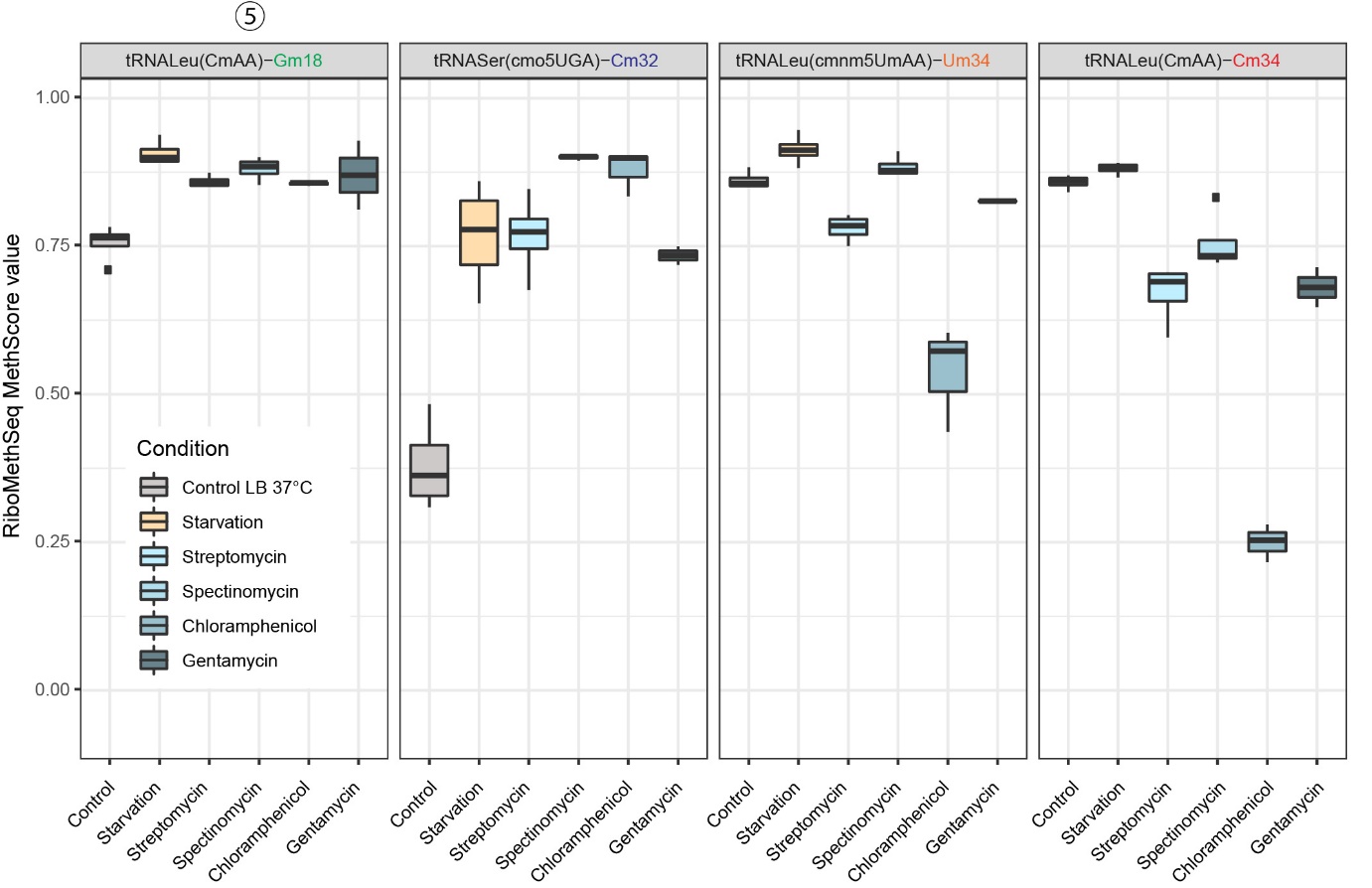


Supp Figure S4C. RiboMethSeq analysis of 2’-O-methylation for selected stress conditions (2 to 4 biological replicates). Panel C - MethScore values for 4 selected positions in *E. coli* tRNAs (Gm18, Cm32, U*m34 and Cm34), observed under standard (control) conditions LB 37°C and under starvation in PBS (starvation) and sub-lethal antibiotic stress. The identity of analyzed tRNA position is shown on the top of each panel, identity of sample is indicated in color and shown on the bottom. Barplot represents mean values (n=2 to 4), dispersion (1st quantile) and outliers (black dots).


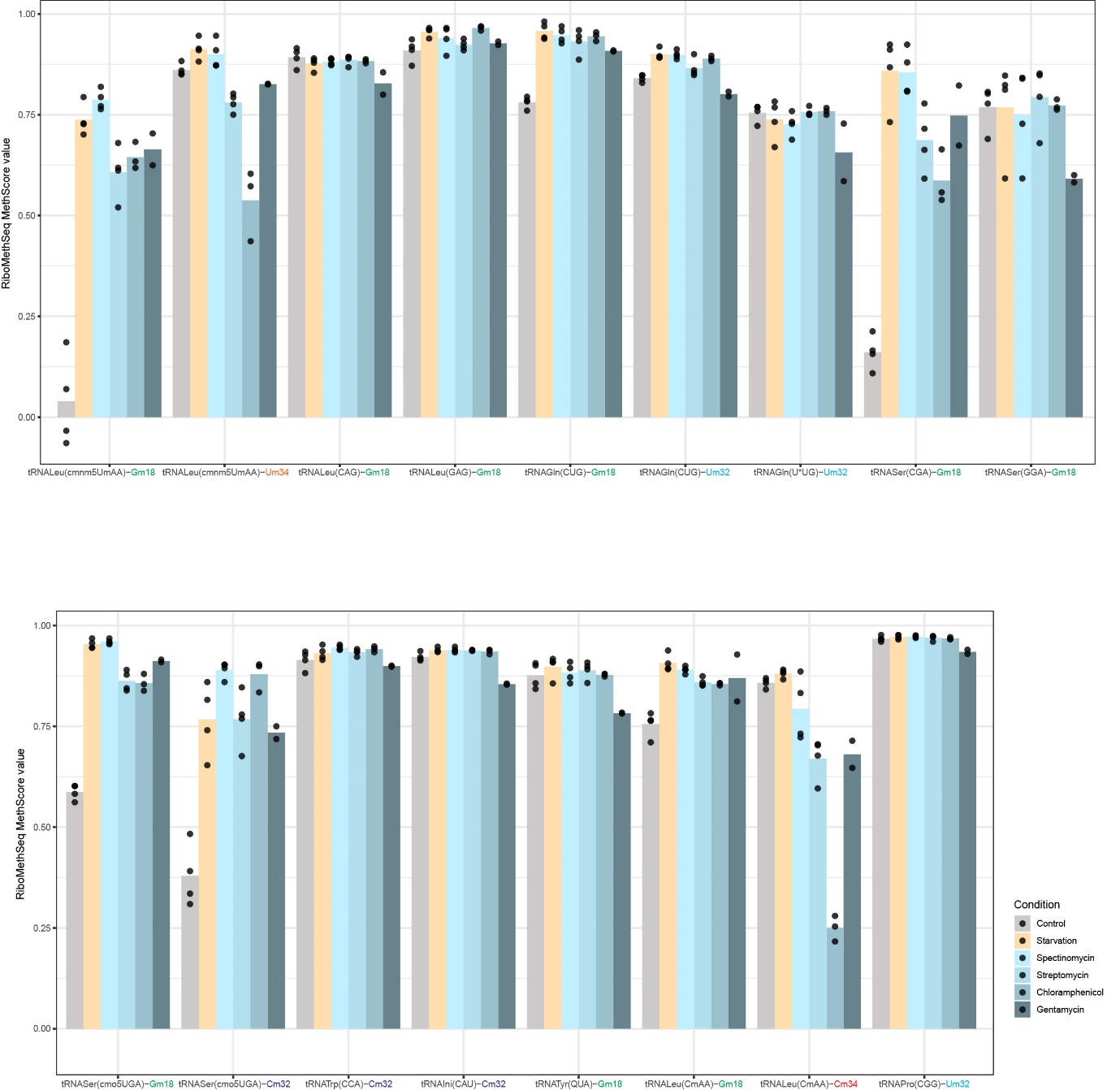


Supp Figure S4D. RiboMethSeq analysis of 2’-O-methylation for selected stress conditions (2 to 4 biological replicates). Panel D - barplot representation of the raw data, showing individual biological replicates for all tested conditions. Color bars correspond to the average values; black dots show measurements for biological replicates.


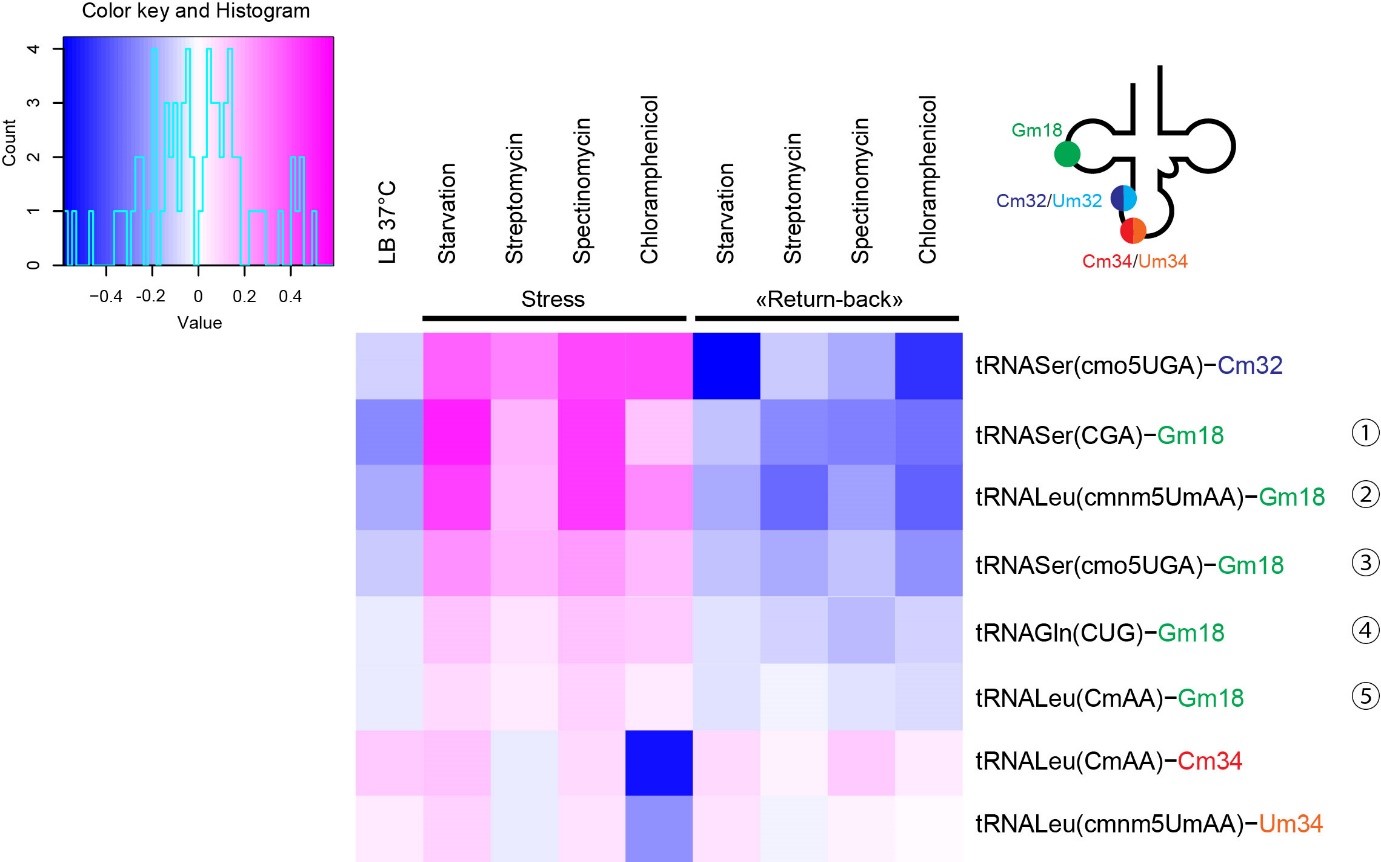


Supp Figure S5. RiboMethSeq analysis of the “return-back” experiment. Bacterial culture was exposed to stress for 24h, aliquot was taken, diluted and grown un to OD_600nm_ ~0.8-0.9. 2’-O-methylation at the selected sites in tRNA was measured. Heatmap of row-normalized MethScore values by position in tRNA (shown on the right) in different stress conditions and control (shown at the bottom). Scale of values is shown in the Color key (top left).


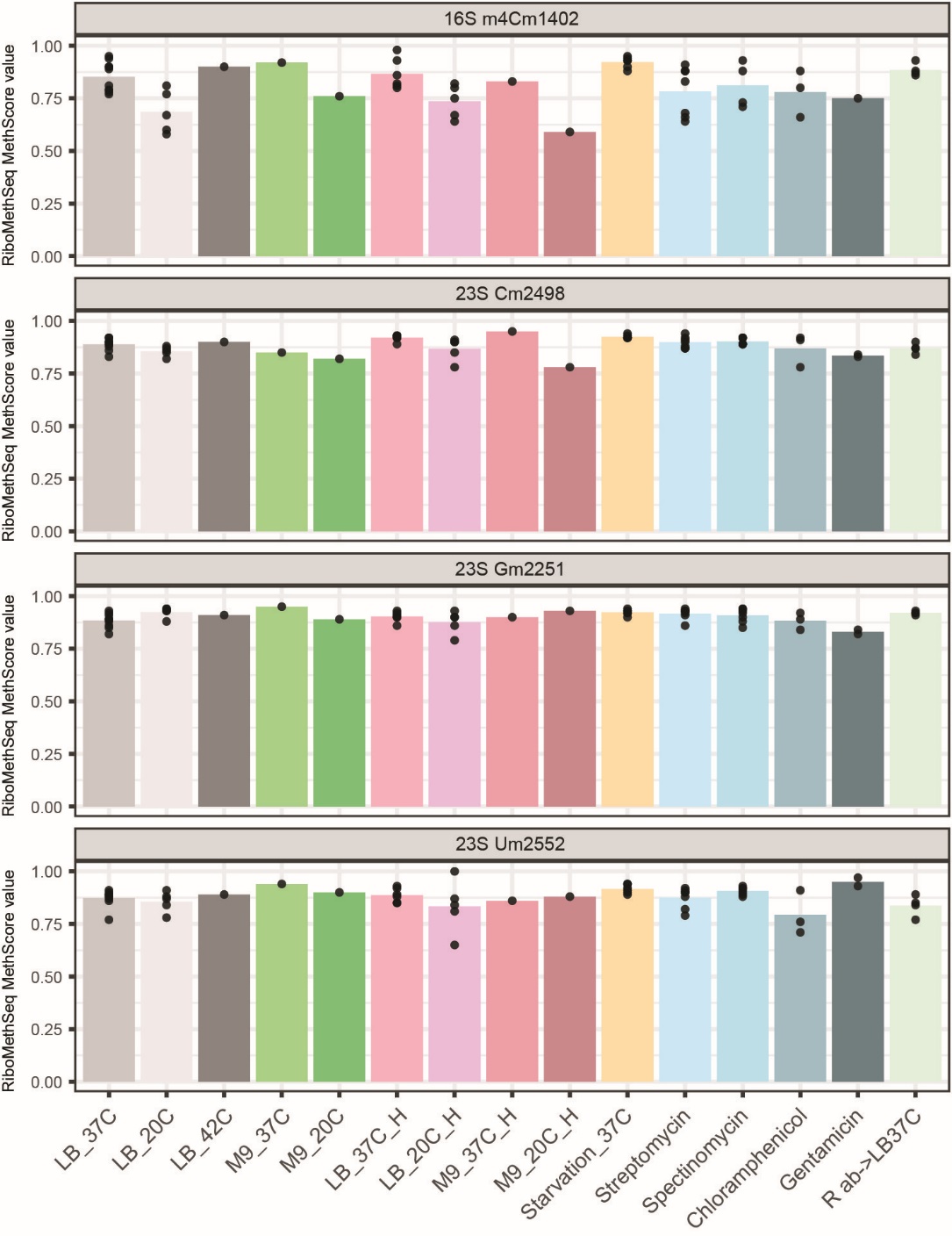


Supp Figure S6. RiboMethSeq analysis of *E. coli* 23S rRNA 2’O-methylation. MethScores are represented for all conditions for the three 2’O-methylations mapped in *E. coli* 23S rRNA: Gm2251, Cm2498 and Um2552.


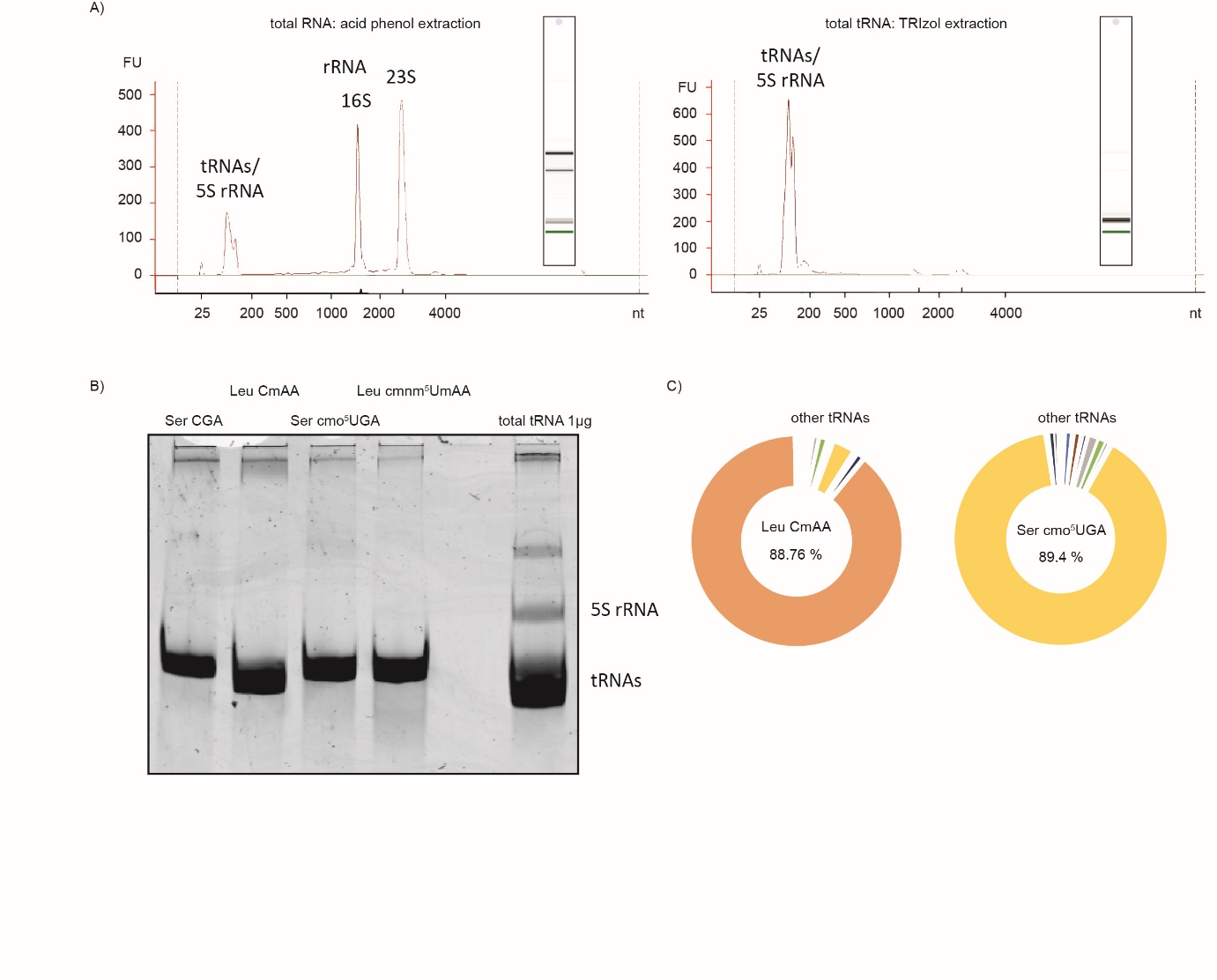


Supp Figure S7 Isolation of individual tRNA species from total *E. coli* tRNA fraction. Panel A - Bioanalyzer 2100 trace of total RNA from *E. coli* (left) and total tRNA fraction from the same source, obtained by direct Trizol extraction. Panel B - purity of individual tRNA species obtained by DNA-oligonucleotide affinity chromatography was assessed by urea-PAGE. Four purified *E. coli* tRNA samples (tRNASer(CGA), tRNALeu(CmAA), tRNASer(cmo^5^UGA) and tRNALeu(cmnm^5^UmAA) were loaded on the gel, in parallel with total tRNA fraction from *E. coli*. Panel C - purity of the extracted tRNA was evaluated by deep sequencing, after RiboMethSeq-like type of library preparation (with tRNA fragmentation). Reads mapping to expected tRNA species represent almost 90% of total reads obtained for the sample.

Supp Figure S8 Knockout of TrmH enhances swarming motility in *E. coli*.

Swarming motility of *E. coli* parental strain and ΔTrmH strain on motility agar. 2 µl of overnight cultures in TB medium were inoculated into semisolid TB agar containing 0.3 % (wt/vol) agar and incubated at 33°C. (A) The photographs show representative images of swarming behavior of *E. coli* parental strain and ΔTrmH strain after incubation for 1 h, 8 h and 24 h. (B) Swarm diameters were measured after 24 h. Scatter plot depicting median swarm diameters, every symbol represents the value for one colony. n=8; *: p-value < 0.05. Statistical values were calculated using Mann-Whitney test.


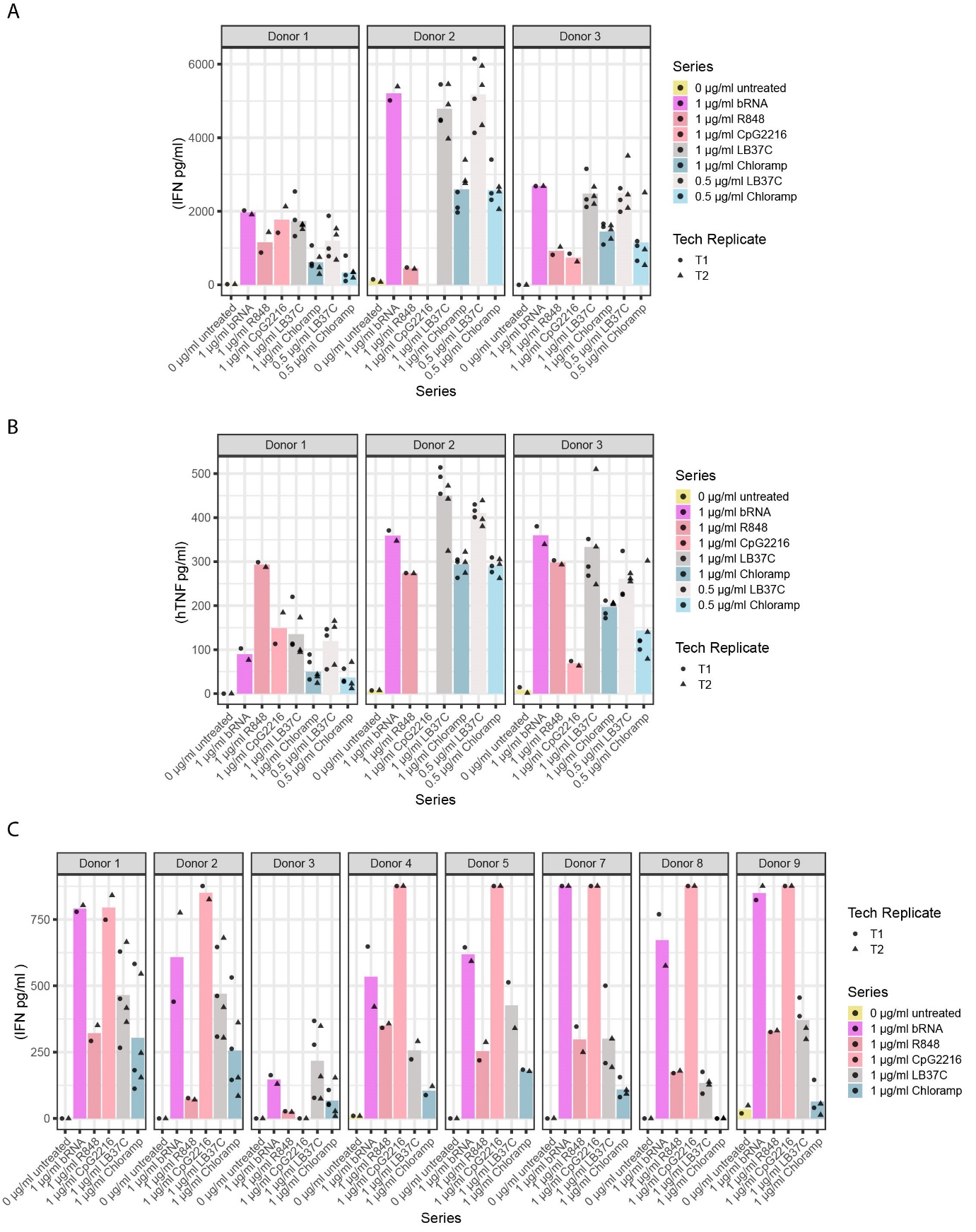


Supp Figure S9 Immunostimulation properties of *E. coli* total tRNA fraction measured using IFNα and TNFα release by human pDC and PBMCs, data represented by individual donor. (A,B) Human pDCs from 3 healthy donors were stimulated with total tRNA from control conditions (LB 37°C) and from chloramphenicol treated *E. coli* lab strain culture at two different concentrations (1 µg/ml and 0.5 µg/ml). Total RNA from *S. aureus* (bRNA), R848 and CpG2216 served as positive control. The IFNα (A) and TNFα (B) release was measured for three biological replicates of tRNA fractions and in two technical replicates for ELISA measurements. (C) Human PBMCs from healthy donors were stimulated with total tRNA from 3 different clinical *E. coli* strains isolated from patient’s urine samples at 1 µg/ml. The *E. coli* strains were cultured under control conditions (LB 37°C) and stress conditions (chloramphenicol treated). Total RNA from *S. aureus* (bRNA), R848 and CpG 2216 served as positive control. The IFNα release was measured for one to three biological replicates of tRNA fractions and in two technical replicates for ELISA measurements.


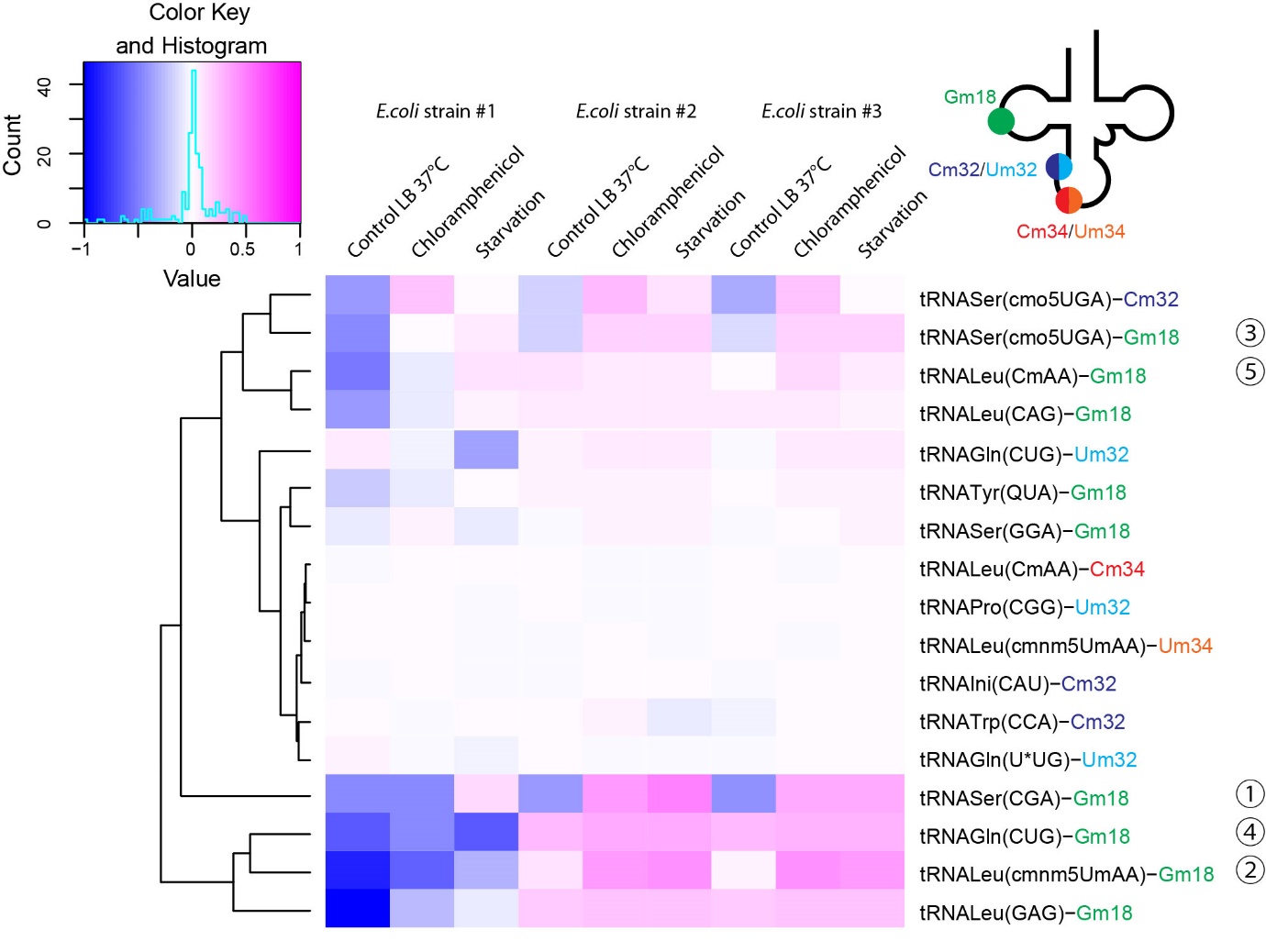


Supp Figure S10 RiboMethSeq analysis of tRNA 2’-O-methylation in clinical *E. coli* isolates. Strains were grown in LB at 37°C, under sublethal chloramphenicol stress (Cam) and in PBS (starvation). Heatmap represents MethScore values for measurable positions of 2’-O-methylation known in *E. coli* tRNAs (positions in grey are excluded). Normalization of MethScore values was done as a difference to the average MethScore for all samples at a given position. Undermethylation compared to average is shown in blue, overmethylation is in pink. Scale of MethScore variation is shown in the Color Key (top left). tRNA identity, anticodon and modified position are indicated on the right. Clustering was done by hclust R function using ward.D2 algorithm.
